# Rapid and scalable personalized ASO screening in patient-derived organoids

**DOI:** 10.1038/s41586-024-08462-1

**Published:** 2025-01-22

**Authors:** John C. Means, Anabel L. Martinez-Bengochea, Daniel A. Louiselle, Jacqelyn M. Nemechek, John M. Perry, Emily G. Farrow, Tomi Pastinen, Scott T. Younger

**Affiliations:** 1 Genomic Medicine Center, Children’s Mercy Kansas City, Kansas City, MO USA; 2 Children’s Mercy Research Institute, Children’s Mercy Kansas City, Kansas City, MO USA; 3 Department of Pediatrics, University of Missouri-Kansas City School of Medicine, Kansas City, MO USA; 4 Department of Pediatrics, University of Kansas Medical Center, Kansas City, KS USA

**Keywords:** Antisense oligonucleotide therapy, Stem-cell differentiation, Reprogramming, Stem-cell biotechnology

## Abstract

Personalized antisense oligonucleotides (ASOs) have achieved positive results in the treatment of rare genetic disease^1^. As clinical sequencing technologies continue to advance, the ability to identify patients with rare disease harbouring pathogenic genetic variants amenable to this therapeutic strategy will probably improve. Here we describe a scalable platform for generating patient-derived cellular models and demonstrate that these personalized models can be used for preclinical evaluation of patient-specific ASOs. We describe protocols for delivery of ASOs to patient-derived organoid models and confirm reversal of disease-associated phenotypes in cardiac organoids derived from a patient with Duchenne muscular dystrophy (DMD) with a structural deletion in the gene encoding dystrophin (*DMD*) that is amenable to treatment with existing ASO therapeutics. Furthermore, we designed novel patient-specific ASOs for two additional patients with DMD (siblings) with a deep intronic variant in the *DMD* gene that gives rise to a novel splice acceptor site, incorporation of a cryptic exon and premature transcript termination. We showed that treatment of patient-derived cardiac organoids with patient-specific ASOs results in restoration of DMD expression and reversal of disease-associated phenotypes. The approach outlined here provides the foundation for an expedited path towards the design and preclinical evaluation of personalized ASO therapeutics for a broad range of rare diseases.

## Abstract (web summary)

A scalable platform for generating patient-specific organoids for testing personalized oligonucleotide therapeutics is described.

## Main

ASOs are short synthetic nucleic acid molecules that, when designed to be complementary to an intracellular mRNA target, can influence RNA processing and/or affect protein expression levels. In the four decades since their initial application in the laboratory, more than a dozen different antisense therapies have been approved in the USA for the treatment of various diseases^2^. The inherent flexibility of a sequence-based targeting modality, in combination with well-established pharmacokinetic and pharmacodynamic properties, make ASOs a particularly attractive strategy for the treatment of genetic disorders. Moreover, the ability to customize ASO designs to individual patients has major implications for personalized therapeutics. For example, personalized ASOs designed against a splice-disrupting transposon insertion within an intron of the *MFSD8* gene in a patient with Batten disease were recently shown to greatly reduce the frequency and duration of disease-associated seizures^1^. Although encouraging, the overwhelming time and cost requirements for the design and preclinical evaluation of personalized ASOs currently precludes this strategy from more widespread adoption.

Induced pluripotent stem (iPS) cells have become powerful tools for modelling developmental processes and dissecting disease pathology^3,4^. Recent advancements in the generation of three-dimensional organoids from patient-derived iPS cells have further expanded the potential of these cellular systems^5^. However, current approaches for establishing patient-derived iPS cell models can be time consuming and costly. These limitations often prevent patient-derived models from reaching their full therapeutic potential with respect to the patients from which they were generated.

We address several of these challenges through the development of a rapid, robust and scalable platform for the generation of patient-derived cell models. We describe an iPS cell reprogramming pipeline that utilizes cryopreserved peripheral blood mononuclear cells (PBMCs), commonly available from previous genetic testing, and requires only 3 weeks to establish iPS cell lines. In addition, we demonstrate that three-dimensional organoid models generated from patient-derived iPS cells recapitulate disease-associated phenotypes that can be reversed using patient-specific ASOs. The approach that we outline, from procurement of patient PBMCs to empirical analysis of ASO effects on organoid function, requires no specialized equipment and less than 6 weeks of hands-on time. We anticipate that implementation of the platform that we describe will lead to the rapid development of preclinical ASO leads for the treatment of many rare genetic diseases.

## Patient-derived iPS cell generation at scale

Our research institute recently launched a rare disease initiative, ‘Genomic Answers for Kids’, which aims to sequence the genomes of 30,000 children impacted by genetic conditions over the course of 7 years^6^. Upon enrolment, patients provided a standard blood draw for genome sequencing and residual PBMCs were cryopreserved for future research use. We reasoned that these patient-specific PBMCs could be a valuable resource for building a biobank of patient-derived cellular models of rare genetic diseases. Realizing this potential, however, requires several technical hurdles to be addressed.

**Fig. 1:**
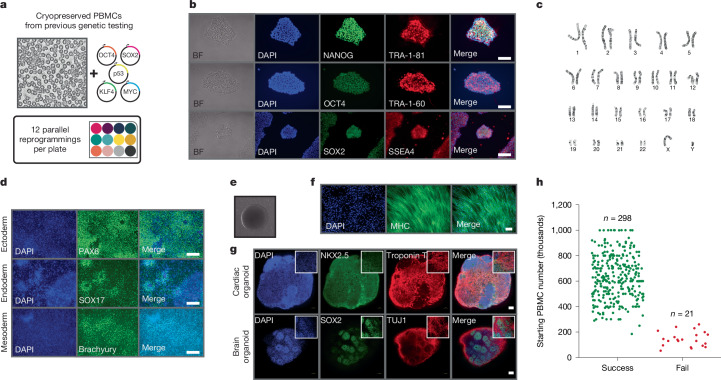
A rapid and scalable platform for the generation of patient-derived cellular models. **a**, Schematic of the iPS cell reprogramming workflow. **b**, iPS cell marker expression in patient-derived iPS cells (patient 1). BF, bright field. **c**, Representative karyotype of patient-derived iPS cells (patient 1). **d**, Differentiation of patient-derived iPS cells (patient 1) into ectoderm, endoderm and mesoderm lineages. **e**, Embryoid body formation using patient-derived iPS cells (patient 1). **f**, Differentiation of patient-derived iPS cells (patient 1) into two-dimensional skeletal muscle. **g**, Differentiation of patient-derived iPS cells (patient 1) into three-dimensional cardiac and brain organoids. **h**, Reprogramming outcomes relative to PBMC input cell counts. Data in **b**,**d**,**f**,**g** are representative of *n* = 3 biologically independent experiments. Scale bars, 100 µm (**b**,**d**,**f**,**g**).

Generating iPS cells from patient cells can be achieved through several different techniques. Viral-based approaches (for example, Sendai virus) can be highly efficient, but the current reagent expense is not amenable to scalability^7^. Episomal plasmid-based approaches are more cost-effective but are less efficient and require more source material^8^. To overcome these limitations, we extracted key features from multiple iPS cell reprogramming strategies, ultimately coalescing these methods into a single basic protocol^9–26^. More specifically, our protocol included a customized reprogramming efficiency cocktail (the GSK inhibitor CHIR99021, the MEK inhibitor PD0325901, the TGFβ–activin–nodal receptor inhibitor SB431542, the ROCK inhibitor Y-27632, sodium butyrate, ascorbic acid and bFGF in ReproTESR) and a centrifugation step to facilitate attachment to Matrigel-coated plates. The advantages of each component of the reprogramming cocktail are outlined in Supplementary Table 1. Using this protocol, we were routinely able to reprogram PBMCs into iPS cells, expand iPS cell lines and cryopreserve patient-derived iPS cell lines into our biobank within 2–3 weeks (Fig. 1a). Moreover, reprogramming reactions were performed in a single well of a 12-well dish, which conveniently enabled 12 independent reprogramming experiments to be performed in parallel (Fig. 1a).

**Extended Data Fig. 1:**
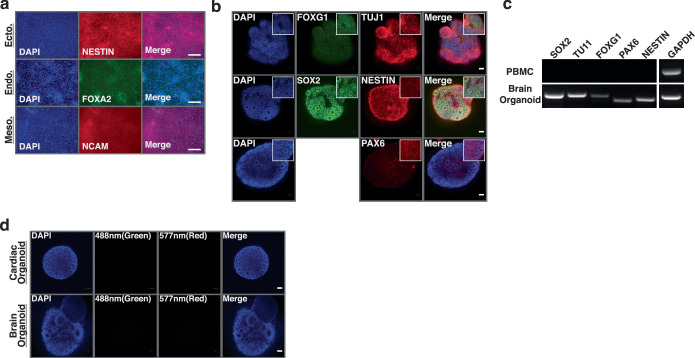
Validation of trilineage differentiation from patient-derived iPSCs. **a)** Differentiation of patient-derived iPSCs (patient 1) into ectoderm, endoderm, and mesoderm lineages. **b)** Neuronal marker expression in patient-derived cerebral organoids (patient 1). **c)** RT-PCR confirmation of neuronal marker expression in patient-derived cerebral organoids (patient 1). **d)** Background staining from secondary antibodies in cardiac and brain organoids. Data in **c** are representative of *n* = 3 biologically independent experiments. Scale bars in **a**, **b**, **d** represent 100 µm.

We performed extensive characterizations of the iPS cell lines generated using our protocol. We confirmed expression of several iPS cell markers (Fig. 1b) and evaluated genome integrity through karyotyping (Fig. 1c). In addition, we confirmed the pluripotency of our iPS cells using trilineage differentiation assays (Fig. 1d and Extended Data Fig. 1a) as well as embryoid body formation (Fig. 1e). We further validated the pluripotency of our iPS cell lines by demonstrating their ability to differentiate into skeletal muscle (Fig. 1f), cardiac organoids (Fig. 1g) and brain organoids (Fig. 1g and Extended Data Fig. 1b,c). To ensure the specificity of antibodies used for immunofluorescence assays, we performed negative control staining using only secondary antibodies and detected no background fluorescence (Extended Data Fig. 1d).

To assess the long-term stability of iPS cell lines generated using our protocol, we recharacterized their pluripotency after 25–30 passages. Late-passage iPS cells retained expression of pluripotency markers (Supplementary Fig. 1a,b). Moreover, late-passage iPS cells continued to form tightly packed colonies (Supplementary Fig. 1c). We did not detect any karyotypic abnormalities in iPS cells after 25 passages (Supplementary Fig. 1d). Furthermore, trilineage differentiation assays confirmed that late-passage iPS cells remained pluripotent (Supplementary Fig. 1e).

**Extended Data Fig. 2:**
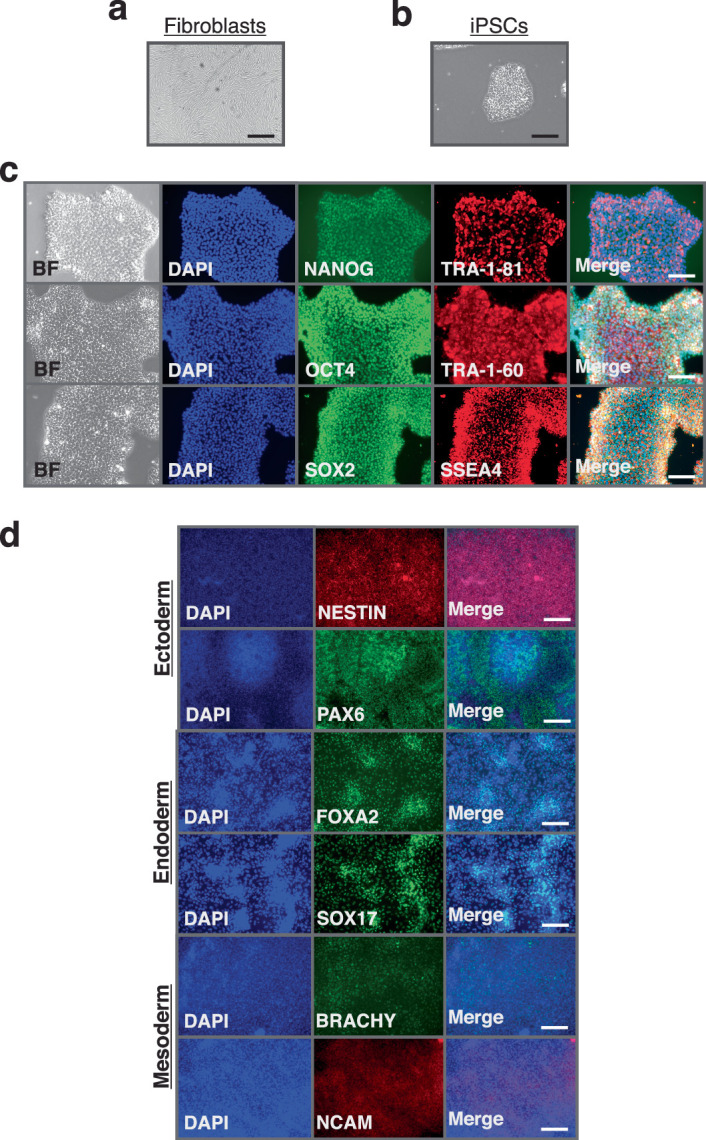
Generating iPSC models from patient fibroblasts. **a)** Patient fibroblasts. **b)** A representative iPSC colony derived from patient fibroblasts. **c)** iPSC marker expression in iPSCs derived from patient fibroblasts. **d)** Differentiation of iPSCs derived from patient fibroblasts into ectoderm, endoderm, and mesoderm lineages. Scale bars in **a**,**b**,**c**,**d** represent 100 µm.

Given the high efficiency and inherent scalability of our reprogramming pipeline, we sought to determine the reliability and robustness of our approach through a campaign of iPS cell reprogramming experiments. Over the course of 6 months, we were able to generate nearly 300 patient-derived iPS cell lines with more than 93% success rate (Fig. 1h). We found that reprogramming outcomes were influenced predominantly by the number of available PBMCs and not by any intrinsic features of a given patient or disease phenotype. More specifically, we were able to generate iPS cells for 100% of patients for which at least 300,000 PBMCs were available (Fig. 1h). Of note, our protocol was also capable of deriving iPS cells from patient fibroblasts (Extended Data Fig. 2a–d). The iPS cell reprogramming protocol that we outline does not require specialized equipment or high-cost reagents and can be easily implemented in any standard research laboratory.

## Delivering ASOs into organoids

Having harnessed the ability to rapidly generate patient-derived cellular models, we next focused our efforts on performing genetic perturbations in these systems. We focused specifically on ASO technology due to their established use as a laboratory reagent and their recent application towards personalized therapeutics^1^. To optimize our ASO delivery strategy, we selected a target gene for which: (1) protein expression could be easily quantified, and (2) inhibition of gene expression would have a visible effect on cellular phenotypes. Cardiac troponin T (encoded by *TNNT2*) is highly expressed in cardiomyocytes and is required for cardiomyocyte contraction^27–29^. We reasoned that inhibition of cardiac troponin T in iPS cell-derived cardiac models would provide an ideal system for evaluating ASO delivery and efficacy.

**Fig. 2:**
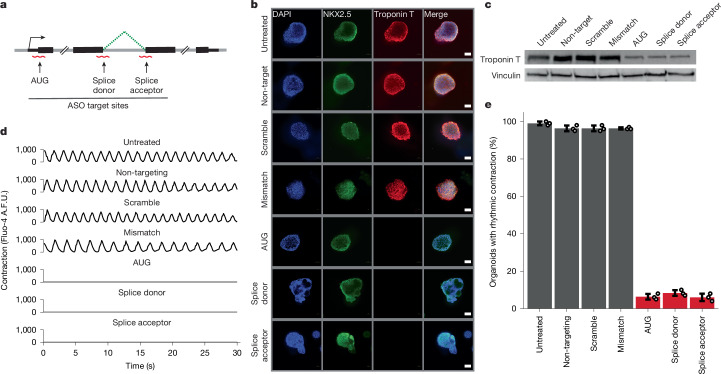
Robust genetic perturbation in organoid systems using ASOs. **a**, Schematic of the design strategy for ASOs targeting cardiac troponin T. **b**, Cardiac troponin T expression in cardiac organoids treated with ASOs. **c**, Western blot confirmation of cardiac troponin T expression in cardiac organoids treated with ASOs. **d**, Contraction of individual cardiac organoids treated with ASOs as determined by intracellular calcium levels. A.F.U., arbitrary fluorescence units. **e**, Summarized contraction of cardiac organoids treated with ASOs. Data in panels **b**,**c** are representative of *n* = 3 biologically independent experiments. Data in panel **e** are presented as mean ± s.d. Error bars in **e** represent standard deviation for *n* = 3 counts of 100 independent organoids. Scale bars, 100 µm (**b**).

To explore a diversity of genetic manipulations, we designed three distinct ASOs targeting different regions of the cardiac *TNNT2* transcript (Fig. 2a). We first designed an ASO targeting the AUG translation start site, anticipating that ASO binding would sterically interfere with ribosome assembly and prevent protein production. Several existing ASO therapeutics function through the modulation of RNA splicing as opposed to direct inhibition of protein translation^30–33^. To ensure that our ASO strategy was capable of influencing RNA splicing, we designed two additional ASOs: one targeting a splice donor site and one targeting a splice acceptor site. We synthesized all ASOs as fully modified 2′-*O*-methyl RNAs.

We next evaluated ASO-mediated gene inhibition in cardiac organoids generated from a commercially available iPS cell line. To ensure the quality of the iPS cell line, we first assessed both pluripotency and genomic stability (Supplementary Fig. 2a–k). We differentiated the iPS cells into contracting cardiac organoids, a 2-week process, followed by transfection with the ASOs. Cardiac organoids treated with a negative control ASO with no complementarity to the human genome expressed high levels of cardiac troponin T (Fig. 2b,c). By contrast, cardiac troponin T expression was substantially reduced in cardiac organoids treated with ASOs targeting the AUG translation start site (Fig. 2b,c). We designed two additional negative control ASOs based on the troponin T AUG-targeting ASO, a scrambled ASO and an ASO containing four evenly spaced mismatches. Neither of these negative control ASOs affected the expression of troponin T (Fig. 2b,c). However, the expression of cardiac troponin T was significantly reduced in cardiac organoids treated with ASOs targeting splice donor or acceptor sites, suggesting that RNA splicing had been altered (Fig. 2b,c).

To quantify the effect of cardiac troponin T inhibition on cardiac organoid function, we evaluated calcium fluctuations using the Fluo-4 AM calcium indicator dye. Cardiac organoids treated with any of the negative control ASOs displayed robust and rhythmic cycles of calcium transients that were indistinguishable from untreated organoids (Fig. 2d and Supplementary Videos 1–4). However, calcium transients were undetectable in cardiac organoids treated with cardiac troponin T-targeting ASOs (Fig. 2d and Supplementary Videos 5–7). Cardiac organoid phenotypes were highly robust, with a loss of rhythmic contraction observed in more than 90% of transfected organoids (Fig. 2e). Together, these data demonstrate that ASOs can achieve robust genetic perturbation in an iPS cell-derived organoid model.

## Evaluating existing ASO therapeutics

**Fig. 3:**
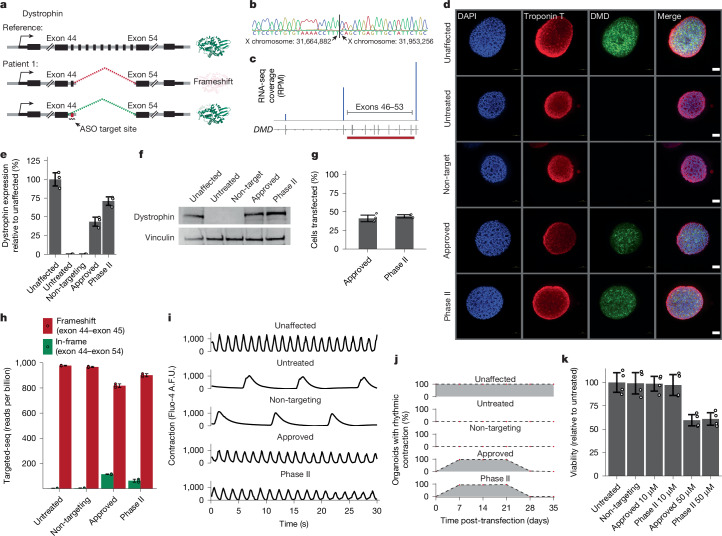
Profiling activity of existing ASO therapeutics in a patient-derived organoid model of disease. **a**, Schematic of the design strategy to profile existing ASO therapeutics for the treatment of DMD. **b**, Chromatogram validating structural deletion within the *DMD* gene in patient 1. **c**, Targeted RNA-seq validating missing exons in the expressed *DMD* transcript in patient 1. RPM, reads per million. **d**, Dystrophin expression in cardiac organoids derived from a patient with DMD (patient 1) treated with ASOs matching the sequence of existing ASO therapeutics. **e**, Quantification of dystrophin expression in cardiac organoids derived from a patient with DMD (patient 1) compared with unaffected cardiac organoids. **f**, Western blot confirmation of dystrophin expression in cardiac organoids derived from a patient with DMD (patient 1) treated with ASOs matching the sequence of existing ASO therapeutics. **g**, Efficiency of ASO transfection in patient-derived cardiac organoids (patient 1). **h**, Relative expression of exon 45-containing versus exon 45-skipped *DMD* transcripts in cardiac organoids derived from a patient with DMD (patient 1) treated with ASOs matching the sequence of existing ASO therapeutics. **i**, Contraction of individual cardiac organoids derived from a patient with DMD (patient 1) treated with ASOs matching the sequence of existing ASO therapeutics. **j**, Time-course analysis of restored contraction (based on visual assessment of contraction) in cardiac organoids derived from a patient with DMD (patient 1) treated with ASOs matching the sequence of existing ASO therapeutics. **k**, Viability of cardiac organoids derived from a patient with DMD (patient 1) treated with ASOs. Data in **d**,**f** are representative of *n* = 3 biologically independent experiments. Data in **e**,**g**,**h**,**k** are presented as mean ± s.d. Error bars in **e**,**h** represent standard deviation for *n* = 3 replicates. Error bars in **g**,**k** represent standard deviation for *n* = 4 replicates. Scale bars, 100 µm (**d**).

Having established methods for characterizing ASOs in iPS cell-derived organoid models, we sought to determine whether these models could be suitable for evaluating the efficacy of ASO therapeutics. Among the iPS cell lines that we generated was one from a patient with DMD (patient 1) with a structural deletion of exons 46–53 of the *DMD* gene, resulting in a frameshift in the *DMD* transcript and a loss of dystrophin protein expression (Figs. 1b–g and 3a, Extended Data Fig. 1a–c and Supplementary Fig. 1a–e). This deletion is amenable to treatment with an FDA-approved ASO therapeutic designed to skip splicing to exon 45, leading to a *DMD* transcript that lacks genetic information but with a restored reading frame^33^. Although DMD is often described in the context of skeletal muscle, most patients ultimately succumb to cardiac failure, making cardiac organoids a highly relevant system for evaluating therapeutic approaches^34,35^. We designed a 2′-*O*-methyl RNA matching the sequence of the FDA-approved therapeutic to profile its activity in a patient-derived organoid model.

**Extended Data Fig. 3:**
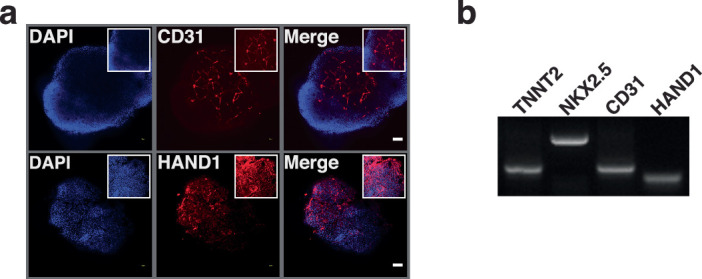
Marker analysis in patient-derived cardiac organoids. **a)** Cardiac marker expression in patient-derived cardiac organoids (patient 1). **b)** RT-PCR confirmation of cardiac marker expression in patient-derived cardiac organoids (patient 1). Data in **b** are representative of *n* = 3 biologically independent experiments. Scale bars in **a** represent 100 µm.

We generated cardiac organoids from unaffected iPS cells as well as from iPS cells derived from patient 1. We validated the resulting cardiac organoids by measuring expression of several cardiac markers (Fig. 1g and Extended Data Fig. 3a,b). We confirmed the presence of the structural deletion in cardiac organoids generated from patient 1 by isolating genomic DNA and performing Sanger sequencing across the region (Fig. 3b). In addition, we performed targeted RNA sequencing (RNA-seq) in the region of the *DMD* transcript affected by the structural deletion and confirmed the absence of exons 46–53 (Fig. 3c). We next evaluated dystrophin expression in cardiac organoids derived from patient 1. In contrast to unaffected cardiac organoids, which express high levels of dystrophin, expression was completely absent in organoids generated from patient 1 (Fig. 3d–f). However, treatment with an ASO matching the sequence of the approved therapeutic restored dystrophin expression to levels comparable with a healthy cardiac organoid (Fig. 3d–f). We also profiled the activity of a second exon 45-skipping ASO that targets a different sequence in the *DMD* transcript and is currently in Phase II clinical trials^36^. Treatment with a 2′-*O*-methyl RNA matching the sequence of the therapeutic in clinical trials also restored dystrophin expression to levels comparable with a healthy cardiac organoid (Fig. 3d–f).

Having demonstrated that exon 45-skipping ASOs are able to restore dystrophin expression in patient-derived cardiac organoids, we sought to determine the molecular efficacy of our experimental approach. We first determined the efficiency of transfection into cardiac organoids by labelling the exon 45-skipping ASOs with a fluorescent tag and performing flow cytometry. We found that approximately 40% of cells in the organoids contained ASOs (Fig. 3g). We next performed targeted RNA-seq on the *DMD* transcript to quantify exon 45 skipping at the level of *DMD* RNA. We determined that exon 45 was absent from approximately 15% of detected transcripts (Fig. 3h).

We next profiled the function of cardiac organoids generated from patient 1 using the Fluo-4 AM calcium indicator dye. Organoids generated from patient 1 displayed weak and arrhythmic patterns of calcium transients (Fig. 3i and Supplementary Videos 8–10). Treatment of patient-derived cardiac organoids with exon 45-skipping ASOs restored the rhythmic calcium fluctuations that we observed in healthy cardiac organoids (Fig. 3i and Supplementary Videos 8, 11 and 12). Organoid contractions were robust up to 21 days post-transfection (Fig. 3j). To ensure that the functional effects of exon 45-skipping ASOs were not resulting from transfection-related toxicity, we evaluated the viability of transfected organoids. Transfecting ASOs at 10 µM, the concentration used throughout this study, had no effect on cell viability (Fig. 3k). These results indicate that patient-derived organoid models can be used to evaluate the efficacy of ASO therapeutics.

To establish the generalized applicability of our approach, we evaluated the ability of exon-skipping ASOs to restore dystrophin expression in alternative contexts. We first generated cardiac organoids using an alternative differentiation protocol before ASO transfection. We observed robust restoration of dystrophin expression following ASO addition, indicating that ASO efficacy is not dependent on a specific differentiation protocol (Supplementary Fig. 3a,b). Likewise, treatment of cardiac organoids generated using the alternative differentiation protocol with exon 45-skipping ASOs restored rhythmic calcium fluctuations (Supplementary Fig. 3c and Supplementary Videos 13–17). We also observed ASO-mediated restoration of dystrophin expression in skeletal muscle derived from patient 1 (Supplementary Fig. 4a,b).

## Evaluating personalized ASO therapeutics

**Fig. 4:**
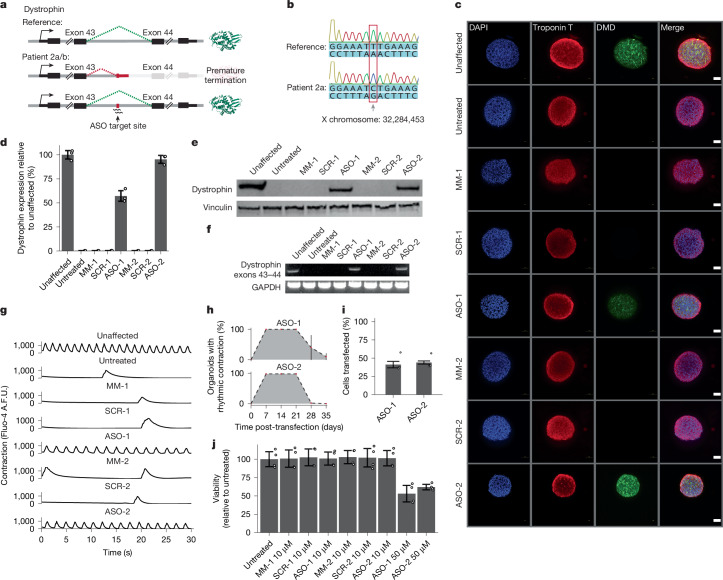
Design and preclinical evaluation of patient-specific ASOs in patient-derived organoids. **a**, Schematic of the design strategy for preclinical evaluation of novel patient-specific ASOs. **b**, Chromatogram validating intronic variant within the *DMD* gene in patient 2a. **c**, Dystrophin expression in cardiac organoids derived from a patient with DMD (patient 2a) treated with patient-specific ASOs. MM, mismatch; SCR, scramble. **d**, Quantification of dystrophin expression in cardiac organoids derived from a patient with DMD (patient 2a) compared with unaffected cardiac organoids. **e**, Western blot confirmation of dystrophin expression in cardiac organoids derived from a patient with DMD (patient 2a) treated with patient-specific ASOs. **f**, RT–PCR validating restored exon 43–44 splicing in cardiac organoids derived from a patient with DMD (patient 2a) treated with patient-specific ASOs. **g**, Contraction of individual cardiac organoids derived from a patient with DMD (patient 2a) treated with patient-specific ASOs. **h**, Time-course analysis of restored contraction (based on visual assessment of contraction) in cardiac organoids derived from a patient with DMD (patient 2a) treated with patient-specific ASOs. **i**, Efficiency of ASO transfection in patient-derived cardiac organoids (patient 2a). **j**, Viability of cardiac organoids derived from a patient with DMD (patient 2a) treated with ASOs. Data in panels **c**,**e**,**f** are representative of *n* = 3 biologically independent experiments. Data in panels **d**,**i**,**j** are presented as mean ± s.d. Error bars in panel **d** represent standard deviation for *n* = 3 replicates. Error bars in panels **i**,**j** represent standard deviation for *n* = 4 replicates. Scale bars, 100 µm (**c**).

**Extended Data Fig. 4:**
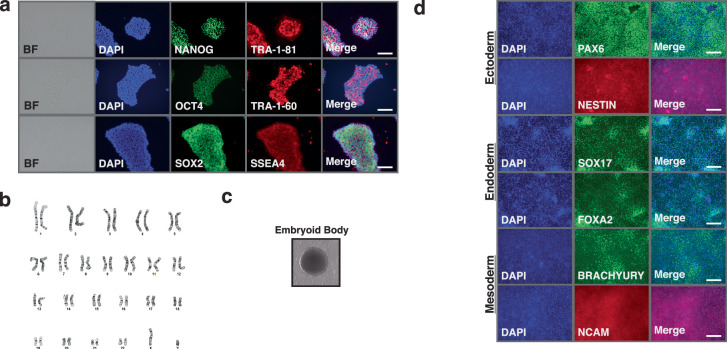
Characterization of patient-derived iPSCs. **a)** iPSC marker expression in patient-derived iPSCs (patient 2a). **b)** Representative karyotype of patient-derived iPSCs (patient 2a). **c)** Embryoid body generated from patient-derived iPSCs (patient 2a). **d)** Differentiation of patient-derived iPSCs (patient 2a) into ectoderm, endoderm, and mesoderm lineages. Scale bars in **a**, **d** represent 100 µm.

**Extended Data Fig. 5:**
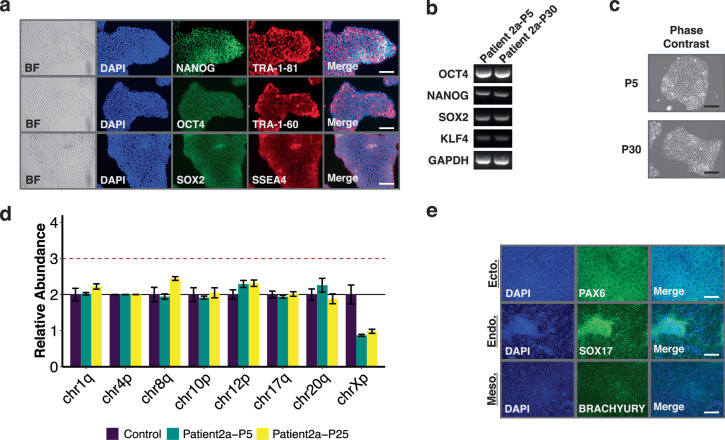
Patient-derived iPSCs remain stable over extended culturing. **a)** iPSC marker expression in patient-derived iPSCs (patient 2a) after 30 passages. **b)** iPSC marker expression in patient-derived iPSCs (patient 2a) after 5 and 30 passages. **c)** Phase contrast imaging of patient-derived iPSC colonies (patient 2a) after 5 and 30 passages. **d)** qPCR analysis of common karyotypic abnormalities in patient-derived iPSCs (patient 2a) after 5 and 25 passages. **e)** Differentiation of late-passage (passage 30) patient-derived iPSCs (patient 2a) into ectoderm, endoderm, and mesoderm lineages. Data in **b** are representative of *n* = 3 biologically independent experiments. Error bars in **d** represent standard deviation for *n* = 3 replicates. Scale bars in **a**, **c**, **e** represent 100 µm.

The ability to tailor the sequence of an ASO to an individual patient is a powerful feature of this therapeutic modality. We next set out to determine whether patient-derived organoid models could be used for the preclinical characterization of novel, patient-specific ASO therapeutics. Included in the cohort of iPS cell lines that we generated were iPS cells from two additional patients with DMD (siblings; patient 2a and patient 2b) who inherited a deep intronic mutation in the *DMD* gene that gives rise to a novel splice acceptor site, incorporation of a cryptic exon and premature transcript termination^37^ (Extended Data Figs. 4a–d and 5a–e). We reasoned that this intronic variant would be an ideal candidate target for an ASO therapeutic and designed two ASOs: both 2′-*O*-methyl RNAs complementary to distinct sequences overlapping the pathogenic variant within the *DMD* intron (Fig. 4a).

**Extended Data Fig. 6:**
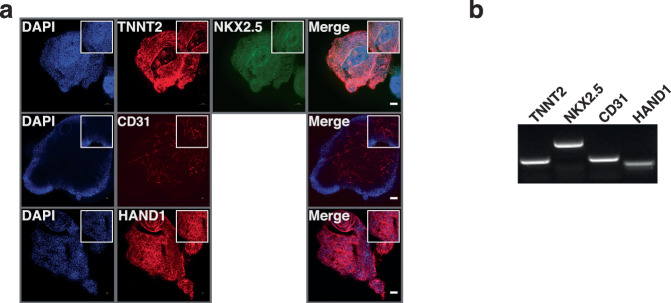
Marker analysis in patient-derived cardiac organoids. **a)** Cardiac marker expression in patient-derived cardiac organoids (patient 2a). **b)** RT-PCR confirmation of cardiac marker expression in patient-derived cardiac organoids (patient 2a). Data in **b** are representative of *n* = 3 biologically independent experiments. Scale bars in **a** represent 100 µm.

To evaluate the efficacy of the personalized ASOs, we generated cardiac organoids from patient 2a and validated expression of several cardiac markers (Extended Data Fig. 6a,b). We confirmed the presence of the deep intronic single-nucleotide variant in cardiac organoids generated from patient 2a by isolating genomic DNA and performing Sanger sequencing across the intronic region (Fig. 4b). As with patient 1, organoids generated from patient 2a lacked detectable dystrophin protein expression (Fig. 4c–e). However, treatment with either of the personalized ASOs that we designed restored dystrophin protein expression to levels comparable with a healthy cardiac organoid (Fig. 4c–e). Restoration of proper *DMD* splicing was further confirmed by RT–PCR with primers spanning the exon 43–44 junction (Fig. 4f). Previous reports have shown that restoration of dystrophin expression at levels equivalent to just 20% of healthy individuals has been sufficient to prevent symptoms of DMD, suggesting that our ASOs are promising preclinical candidates^38,39^.

**Extended Data Fig. 7:**
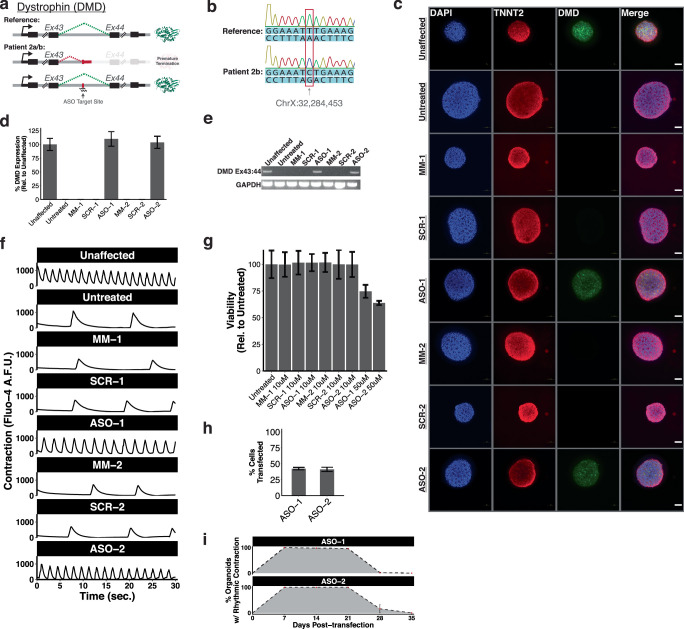
Design and preclinical evaluation of patient-specific ASOs in patient-derived organoids. **a)** Schematic of design and delivery strategy for preclinical evaluation of novel patient-specific ASOs. **b)** Chromatogram validating intronic variant within the DMD gene in patient 2b. **c)** Dystrophin expression in DMD patient-derived cardiac organoids (patient 2b) treated with patient-specific ASOs. **d)** Quantification of DMD expression in DMD patient-derived cardiac organoids (patient 2b) as compared to unaffected cardiac organoids. **e)** RT-PCR validating restored exon 43:44 splicing in DMD patient-derived cardiac organoids (patient 2b) treated with patient-specific ASOs. **f)** Contraction of individual DMD patient-derived cardiac organoids (patient 2b) treated with patient-specific ASOs. **g)** Viability of DMD patient-derived cardiac organoids (patient 2b) treated with ASOs. **h)** Efficiency of ASO transfection in patient-derived cardiac organoids (patient 2b). **i)** Time course analysis of restored contraction in DMD patient-derived cardiac organoids (patient 2b) treated with patient-specific ASOs. Data in **e** are representative of *n* = 3 biologically independent experiments. Data in **d**, **g**, **h** are presented as mean +/− SD. Error bars in **d** represent standard deviation for *n* = 3 replicates. Error bars in **g**, **h** represent standard deviation for *n* = 4 replicates. Scale bars in **c** represent 100 µm.

**Extended Data Fig. 8:**
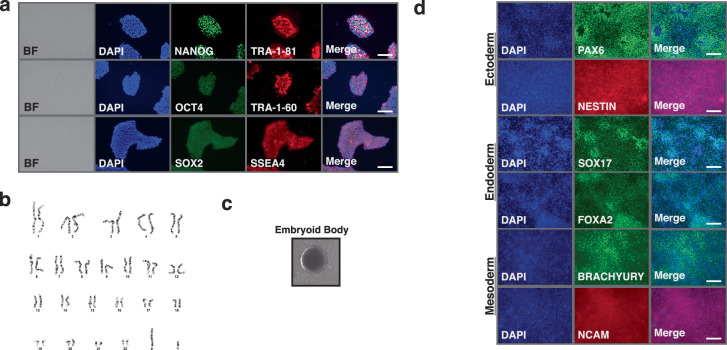
Characterization of patient-derived iPSCs. **a)** iPSC marker expression in patient-derived iPSCs (patient 2b). **b)** Representative karyotype of patient-derived iPSCs (patient 2b). **c)** Embryoid body generated from patient-derived iPSCs (patient 2b). **d)** Differentiation of patient-derived iPSCs (patient 2b) into ectoderm, endoderm, and mesoderm lineages. Scale bars in **a, d** represent 100 µm.

**Extended Data Fig. 9:**
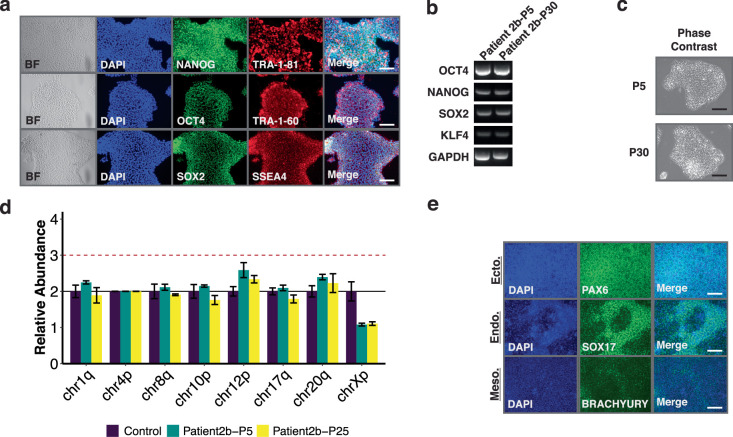
Patient-derived iPSCs remain stable over extended culturing. **a)** iPSC marker expression in patient-derived iPSCs (patient 2b) after 30 passages. **b)** iPSC marker expression in patient-derived iPSCs (patient 2b) after 5 and 30 passages. **c)** Phase contrast imaging of patient-derived iPSC colonies (patient 2b) after 5 and 30 passages. **d)** qPCR analysis of common karyotypic abnormalities in patient-derived iPSCs (patient 2b) after 5 and 25 passages. **e)** Differentiation of late-passage (passage 30) patient-derived iPSCs (patient 2b) into ectoderm, endoderm, and mesoderm lineages. Data in **b** are representative of *n* = 3 biologically independent experiments. Error bars in **d** represent standard deviation for *n* = 3 replicates. Scale bars in **a**, **c**, **e** represent 100 µm.

**Extended Data Fig. 10:**
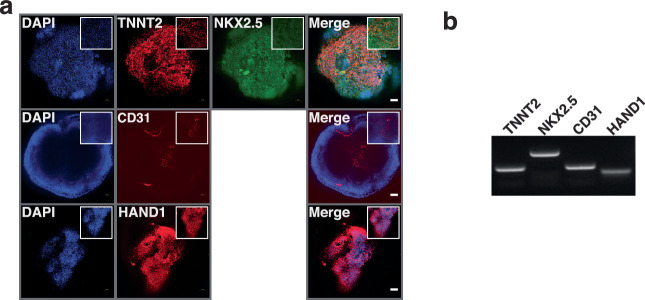
Marker analysis in patient-derived cardiac organoids. **a)** Cardiac marker expression in patient-derived cardiac organoids (patient 2b). **b)** RT-PCR confirmation of cardiac marker expression in patient-derived cardiac organoids (patient 2b). Data in **b** are representative of *n* = 3 biologically independent experiments. Scale bars in **a** represent 100 µm.

Of note, cardiac organoids treated with personalized ASOs displayed rhythmic calcium fluctuations identical to those of a healthy cardiac organoid (Fig. 4g and Supplementary Videos 18–25). As with previous experiments, organoid contractions remained robust up to 21 days post-transfection (Fig. 4h). Likewise, flow cytometry analysis of transfected organoids indicated that approximately 40% of cells in the organoids contained the personalized ASOs (Fig. 4i). Z-stack analysis of transfected organoids revealed that DMD restoration was highest in the outermost layers, suggesting that cells on the surface of the organoid are more amenable to transfection (Supplementary Fig. 5). ASO transfection had no effect on cell viability when introduced at 10 µM concentrations (Fig. 4j). Personalized ASOs also restored dystrophin expression in cardiac organoids generated using the alternative differentiation protocol (Supplementary Fig. 6a–c) as well as in skeletal muscle derived from patient 2a (Supplementary Fig. 7a–c). We obtained indistinguishable experimental results when evaluating the efficacy of the personalized ASOs in cardiac organoids generated from patient 2b (Extended Data Figs. 7–10, Supplementary Figs. 8 and 9 and Supplementary Videos 33–47). In summary, these data establish patient-derived organoid models as powerful systems for the design and characterization of personalized ASO therapeutics.

## Discussion

The ability of ASO therapeutics to be customized to individual patients makes their use an appealing strategy for personalized medicine. The recent demonstration that patient-specific ASOs can greatly improve disease-associated phenotypes further supports the application of this technology towards the treatment of rare diseases caused by genetic factors amenable to an ASO-based approach^1^. However, for the full potential of personalized ASOs to be realized, an expedited process for the preclinical characterization of candidate ASOs is required. We have developed a rapid, robust and scalable platform for generating patient-derived cellular models and have validated the utility of these models in the evaluation of patient-specific ASOs. Although substantial regulatory considerations remain with regards to the approval of personalized ASOs for use in the clinic, the platform that we have described will facilitate widespread preclinical development of personalized ASOs. Increases in the number of prospective patient-specific ASOs will encourage a modernized assessment of the approval process for this unique class of therapeutics.

Patient-derived organoid systems provide a powerful alternative to the use of animal models in the process of drug development. Preclinical studies involving animal models require an average of 6–8 years to complete^40,41^. However, the lifespan for 30% of children born with a rare disease is less than 5 years of age^42^. As illustrated in this study, patient-derived organoid models recapitulate disease phenotypes and can be generated in less than 2 months. Although this study has described the use of patient-derived organoids in the evaluation of personalized ASOs, these systems can be adapted for a broad range of drug development approaches (for example, high-throughput compound screening). Moreover, organoids mimic many key features of human development and offer model systems for exploring diverse aspects of disease biology. As an example, a subset of the cardiac organoids generated in this study contained complex chamber structures that may provide insight into heart development (Supplementary Fig. 10a,b). Although this study has focused largely on the preclinical evaluation of personalized ASOs in cardiac organoids, we have confirmed the ability of these ASOs to restore dystrophin expression in patient-derived brain organoids, further demonstrating the versatility of our platform (Supplementary Fig. 11a–e).

In conclusion, the data described here demonstrate reduction to practice for the use of patient-derived organoid models in the evaluation of personalized therapeutics. The methods and protocols generated in this study are accessible and can be implemented in any standard research laboratory without the need for specialized equipment or high-cost reagents. The widespread ability to generate patient-derived cellular systems will have a substantial effect on the understanding of disease mechanisms as well as potential therapeutic avenues for the treatment of many rare diseases.

## Methods

### Patient consent

Patients profiled in this study are participants in the Genomic Answers for Kids (GA4K) program at the Children’s Mercy Research Institute (https://www.childrensmercy.org/childrens-mercy-research-institute/studies-and-trials/genomic-answers-for-kids/). Potential GA4K participants are informed of the program by clinicians in our health system. Participants met with a member of the study team to ensure any questions they had were answered as a part of the informed written consent into GA4K. Participants did not receive any form of compensation. Genome sequencing data collected through GA4K has been de-identified and deposited in dbGaP (accession: phs002206.v2.p1). Biospecimens collected through GA4K cannot be distributed as general research reagents, but can be shared in instances directly related to achieving diagnoses. The Institutional Review Board of Children’s Mercy Research Institute gave ethical approval for this work (studies #00002465 and #11120514). All methods were carried out in accordance with relevant guidelines and regulations.

### Generation of patient-derived iPS cell lines

Control human iPS cell line (WTC11 background) was obtained from Coriell Institute (GM25256; Supplementary Table 2). Patient iPS cells were generated from PBMCs. Cryopreserved PBMCs were thawed and cultured in StemSpan SFEM II (9605, StemCell Tech) supplemented with StemSpan erythroid expansion supplement (2692, StemCell Tech) and 1X antibiotic–antimycotic (15240062, Gibco) for 2–5 days before reprogramming using episomal plasmids. Episomal plasmids expressing OCT3/4, SOX2, KLF4, L-MYC, LIN28, p53 and EBNA1 (pCE-mP53DD (41856), pCXB-EBNA1 (41857), pCE-HUL (41855), pCE-hsk (41814), pCE-hOCT3/4 (41813; all Addgene) were introduced into PBMCs by electroporation using the Lonza 4D-Nucleofecter. The nucleofected PBMCs were transferred to a Matrigel (354277, Corning)-coated plate and maintained in StemSpan SFEM II plus 10 μM ROCK inhibitor. Two days after nucleofection stem cell media 1 (SCM1) composed of ReproTeSR (5926, StemCell Tech), 10 μM ROCK inhibitor (Y-27632 dihydrochloride; 1254, Tocris), 0.5 μM PD0325901 (72184, StemCell Tech), 6 μM CHIR99021 (72054, StemCell Tech), 2 μM SB431542 (72234, StemCell Tech), 250 μM sodium butyrate (72242, StemCell Tech), 50 μg ml^−1^ ascorbic acid (A8960, Sigma), 10 ng ml^−1^ bFGF (78003, StemCell Tech) and 1X antibiotic–antimycotic were added to each well without removing any media, centrifuged at 50*g* for 30 min and placed back in the incubator for 2 days. After 2 days, media were removed and fresh SCM1 was added. The next day, SCM1 was added without removing media; the following day, all media were removed and fresh SCM1 was added. This was repeated for 1 week. After week 1, media were changed to SCM2 composed of SCM1 minus SB431542, and media were changed exactly as described for SCM1. iPS cell colonies appeared 5–7 days after nucleofection. Once iPS cell colonies appeared, they were allowed to grow in mTeSR1 media supplemented with a cocktail mix of 400 nM sodium butyrate and 100 μg ml^−1^ ascorbic acid. Once colonies were established, ReLeSR (5872, StemCell Tech) was used to select colonies. Standard timeline for reprogramming and expansion was 2–3 weeks for optimal conditions. A summary of iPS cell lines generated from patients with DMD is shown in Supplementary Table 2.

### Cell culture

All iPS cell lines were cultured in mTeSR1 medium on Matrigel-coated flasks in an incubator at 37 °C at 5% CO_2_ until 60–80% confluency was reached, at which point cells were split using ReLeSR.

### ASO design

All ASO molecules used were synthesized (100-nmol scale) at Integrated DNA Technologies with 2′-*O*-methyl modifications (Supplementary Table 3). Troponin-targeted ASOs were designed to overlap either the AUG translation start site, an early splice donor site or an early splice acceptor site based on the *TNNT2* transcript structure annotated in GENCODE Release 43. For *TNNT2-*knockdown experiments, we synthesized a scrambled control based on the AUG-targeting ASO in which the sequence was randomly scrambled but nucleotide composition retained. We also synthesized a mismatched control based on the AUG-targeting ASO containing four nucleotide changes evenly spaced across the sequence (GC content retained). For DMD experiments with existing therapeutics, we synthesized fully modified 2′-*O*-methyl RNAs matching the sequences of the FDA approved and Phase II clinical trial compounds. As a negative control for these experiments, we synthesized an ASO with no complementarity to the human genome. For DMD experiments with personalized ASOs, we designed two distinct ASOs targeting sequences that overlap the intronic single-nucleotide variant detected in patients 2a and 2b. For each personalized ASO, we designed a distinct scrambled control in which the ASO sequence was randomly scrambled but nucleotide composition retained. In addition, we designed distinct mismatched controls containing four nucleotide changes evenly spaced across the ASO sequences (GC content retained). The sequences of all ASOs used in this study are shown in Supplementary Table 3.

### ASO delivery

ASOs were added at a final concentration of 10 µM, which was introduced with the transfection reagent TransIT-TKO (MIR2250, Mirus). For example, TransIT-TKO was added to Opti-MEM (31985062, Thermo Fisher Scientific) and mixed gently by pipetting up and down. For every 100 µl of the Opti-MEM-ASO mix, 3 µl of TransIT-TKO was used. The ASOs were then added to the Opti-MEM-TransIT-TKO mix at the desired concentration and mixed gently by pipetting and incubated at room temperature for 45 min. After 45 min, the ASO transfection mix was added dropwise and gently shaken to evenly distribute the ASOs. ASOs were added to cardiac organoids 14–22 days after the start of differentiation, and media were changed 48 h later.

### iPS cell–cardiac organoid differentiation

To differentiate iPS cells into cardiac organoids, cells were dissociated using Accutase (A1110501, Thermo Fisher Scientific) for embryoid body formation. After dissociation, cells were centrifuged at 300*g* for 3 min and resuspended in mTeSR1 medium (85850, StemCell Tech) containing 10 µM ROCK inhibitor (Y-27632 dihydrochloride; 1254, Tocris). iPS cells were counted and seeded at 5,000 cells per well in a 96-well ultra-low binding, U-shaped-bottom microplate (4515, Corning) or 5,000 cells per microwell when using the AggreWell 800 plates (34825, StemCell Tech). The plate was centrifuged at 100*g* for 3 min and placed in an incubator at 37 °C at 5% CO_2_. After 3–5 days, embryoid bodies were ready for cardiac organoid differentiation. Embryoid bodies have round and smooth edges when they are ready for differentiation. The cardiac differentiation protocol was based on a previously reported small-molecule differentiation method with modifications for use in cardiac organoid differentiation^43–45^. Cardiac differentiation was started by the addition of cardiac differentiation media (CDM; RPMI 1640/B-27; 11875119, A1895601, Fisher), minus insulin containing CHIR99021 (72054, StemCell Tech) at a final concentration of 6 µM. After 2 days, media were changed to CDM, minus insulin containing IWP2 (3533, Tocris) at a final concentration of 5 µM for 2 days. After 2 days, media were changed to CDM, minus insulin for an additional 2 days followed by the addition of maintenance media (RPMI 1640/B27 with insulin; 17504044, Fisher). Media were changed every 2–4 days with maintenance media for the duration of the experiment. The alternative cardiac organoid differentiation protocol was performed as previously described^46^.

### Immunofluorescence

Cardiac organoids were fixed in 4% paraformaldehyde solution (252549, Sigma) overnight at 4 °C. Fixation was followed by washes in 1X PBS (D8537, Sigma) and permeabilization using 1X PBS with 0.2% Triton X-100 (X100, Sigma) for 30 min at room temperature. After permeabilization, blocking was performed overnight at 4 °C in blocking solution (1X PBS, 5% FBS (10082147, Thermo Fisher Scientific), 0.2% Triton X-100 and 2.5% BSA (A9418, Sigma)). The next day, cardiac organoids were washed 3 × 3 min in 1X PBS containing 0.1% BSA followed by the addition of primary antibody solution for 2–3 days at 4 °C. Primary antibody exposure was followed by 5 × 3 min washes and incubation with secondary antibodies for 24 h at 4 °C in the dark. After secondary antibody incubation, cardiac organoids were washed 5 × 3 min in 1X PBS. After secondary incubation, DAPI (AB228549, Abcam) was added at 1:1,000 for 15 min at room temperature, followed by three washes with 1X PBS. Antibody solution was 1:10 of blocking solution to 1X PBS. Primary antibodies were used at 1:100 dilutions (see Supplementary Table 4). Secondary antibodies (goat anti-rabbit Alexa Fluor 488 and goat anti-mouse Alexa Fluor 594; A11034 and A11032, Fisher) were used at 1:400. After immunostaining, organoids were incubated with fructose–glycerol clearing solution (60% glycerol and 2.5 M fructose; 2 h at room temperature followed by overnight at 4 °C) to optically clear organoids before imaging. Organoids were imaged using the Nikon W1 spinning disk confocal microscope.

### Calcium imaging

Cardiac organoids were loaded with 10 µM Fluo-4 AM (F14217, Thermo Fisher Scientific) with 1.25 mM probenecid (P36400, Fisher) and 0.02% pluronic F-127 (P6867, Fisher) added directly to the maintenance media (RPMI 1640/B27 with insulin) for 45 min at 37 °C. The cardiac organoids were washed, and maintenance media were added back and incubated at 37 °C for an additional 30 min before responses were measured using a fluorescence microscope (Keyence BZ-X810) using the FITC filter set. Quantification of cardiac contraction was performed using Musclemotion, a free open-source software for ImageJ.

### Trilineage differentiation

Directed differentiation into all three germ layers (endoderm, mesoderm and ectoderm) was achieved using the STEMdiff Trilineage Differentiation Kit (05230, StemCell Technologies) following the manufacturer’s instructions. In brief, iPS cells were harvested using ReLeSR and single cells were generated by gentle pipetting up and down. This was followed by counting and seeding with recommended cell densities onto Matrigel-coated 24-well plates. Cells were maintained in lineage-specific medium with daily medium changes until day 5 for mesoderm and endoderm differentiation, and until day 7 for ectoderm differentiation. Immunofluorescence staining was performed to assess differentiation to the ectoderm (nestin and PAX6), endoderm (FOXA2 and SOX17) and mesoderm (Brachyury and NCAM).

### Karyotyping

Genomic integrity was assessed by karyotyping with a standard 20 iPS cell analysis. Karyotype services were provided by the Children’s Mercy Kansas City Hospital Genetics laboratory. In addition, the hPSC Genetic Analysis Kit (07550, Stem Cell Technologies) was used to check chromosome abnormalities following the manufacturer’s instructions.

### Cell viability

Cardiac organoid viability was determined using the CellTiter-Glo 3D Cell Viability Assay kit (G9682, Promega) following the manufacturer’s instructions.

### Fluorescently labelling ASOs

ASOs were labelled using the Label IT Nucleic Acid Labeling Kit MFP488 (MIR7125, Mirus) following the manufacturer’s instructions. In brief, the labelling reaction was assembled according to instructions, and was incubated at 37 °C for 3 h followed by purification using G50 microspin purification columns and transfected into cardiac and brain organoids as described above.

### Flow cytometry

Transfection efficiency was assessed by flow cytometry using fluorescently labelled ASOs. Three days after organoid transfection, organoids were dissociated using TrypLE Express (126040132, Thermo Fisher). Organoids were washed once with 1X PBS and then incubated in TrypLE solution at 37 °C for 30 min. Subsequently, organoids were dissociated into single cells by gentle pipetting. Cells were then centrifuged down to remove TrypLE and washed once with 1X PBS (300*g* for 3 min). Pellets were resuspended in 1X PBS supplemented with 2% FBS and 4 mM EDTA in a final volume of 300 µl and stained with 7AAD for 15 min at 4 °C in the dark. Cells were washed twice with PBS–FBS–EDTA and then analysed on a BD Fortessa X-20 flow cytometer. The fraction of ASO-containing cells was measured by gating on total nucleated cells (forward scatter/side scatter), live cells (7AAD negative) and then the percentage of fluorescence-positive cells in the FITC channel. An untransfected control was used to subtract baseline fluorescence intensity. Individual flow cytometry analyses are shown in Supplementary Fig. 12.

### RT–PCR

Total RNA was extracted using an RNeasy Mini Kit (74104, Qiagen) according to the manufacturer’s instructions. RNA was treated with DNAse I, eluted in 50 µl RNase-free water and quantified using a Qubit-4 fluorometer (Invitrogen) or NanoDrop One (Thermo Scientific). A total of 1 µg RNA was reverse transcribed using the SuperScript III First-Strand Synthesis System for RT–PCR (18080-051, Invitrogen) according to the manufacturer’s instructions, and 2 µl was used in PCRs. Amplification of cDNA was carried out with Ex Taq DNA polymerase (RR001A, Takara). The sequences of all primers used in this study are shown in Supplementary Table 5. Uncropped agarose gel scans are shown in Supplementary Fig. 13.

### Western blot

Cardiac organoids were prepared in RIPA lysis buffer (89900, Thermo Fisher), and supernatant was transferred to a fresh 1.5-ml tube. Total protein was quantified using the Pierce BCA Protein Assay Kit (23227, Thermo Fisher) following the manufacturer’s instructions. Thirty micrograms of total protein were resolved on a 3–8% Tris Acetate Criterion XT Precast gel (3450129, Bio-Rad) for DMD or a 4–15% Criterion TGX Precast gel (5671083, Bio-Rad) for cardiac trophin T and vinculin. HiMark Pre-stained Protein ladder (Life Technologies) was used as a size standard for DMD or Precision Plus Protein Dual Color Standards (161-0374, Bio-Rad) for cardiac trophin T and vinculin. The gels were run at 50 V for 15 min followed by 150 V for 1.5 h and then transferred to a 0.4-μm polyvinylidene fluoride membrane (162-0262, Bio-Rad) at 30 V for 6 h at 4 °C. Membranes were blocked in blocking buffer (1X PBS with 0.05%Tween-20 and 5% skim milk) for 1 h at room temperature. An overnight incubation at 4 °C with primary antibody (DMD (1:100), cardiac trophin T (1:200) and viniculin (1:5,000)) was carried out followed by three washes in 1X PBS–0.05% Tween-20 (PBS-T). The membranes were then incubated with corresponding secondary antibody (1:5,000), either goat anti-mouse IRDye800CW (926-32210, LI-COR) or goat anti-rabbit IRDye800CW (926-32211, LI-COR). Western blot results were visualized on an iBright 1500. Uncropped western blot scans are shown in Supplementary Fig. 13.

### Mutation site verification

The genomic DNA from iPS cell lines were extracted using the Nucleospin Blood Kit (740951.50, Macherey-Nagel) and amplified. PCR samples were purified using the QIAquick PCR Purification Kit (28106, Qiagen). All product sizes were confirmed on a 2% agarose gel before Sanger sequencing (Genewiz/Azenta Life Sciences) and Amplicon-Ez Next Generation sequencing (Genewiz/Azenta Life Sciences).

### Amplicon sequencing

For visualization, raw sequencing data were aligned to the human genome using STAR with default settings and GENCODE Release 43 as the reference transcriptome^47^. To quantify ASO-mediated exon skipping, sequencing reads containing exon 44–45 junctions or exon 44–54 junctions were enumerated and normalized to the total number of sequencing reads for the sample.

### Plasmids: skeletal muscle differentiation

LV-TRE-WT human MyoD-dsRedExpress2 was a gift from C. Gersbach (Addgene plasmid #60628).

### Lentivirus production: skeletal muscle differentiation

HEK293T cells (American Type Culture Collection) were seeded (1 × 10^6^ cells per well) in a six-well dish and allowed to attach for 24 h. A transfection mixture of 8.25 µl TransIT LT-1 reagent (Mirus Bio) with psPAX2 vector DNA (11260, Addgene; 1,250 ng), pMD2.G vector DNA (11259, Addgene; 125 ng) and LV-TRE-WT human MyoD-T2A-dsRedExpress2 DNA (96918, Addgene; 1,250 ng) was mixed and the volume brought up with OptiMEM. The transfection mixture was allowed to incubate for 30 min at room temperature and added to the cells. Lentiviral supernatant was collected 48 h post-transfection.

### iPS cell–skeletal muscle differentiation

Skeletal muscle differentiation was based on previous published methods^19^. iPS cells used for skeletal muscle differentiation were maintained in growth medium containing Dulbecco’s modified eagle’s medium (DMEM)/F12 (21041025, Thermo Fisher), 20% knockout serum replacement (10828028, Thermo Fisher), 1% MEM non-essential amino acids (11140050, Thermo Fisher), 2 mM Glutamax (35050079, Thermo Fisher), 100 mM β-mercaptoethanol (21985023, Thermo Fisher) and 10 ng ml^−1^ bFGF (78003.1, StemCell Tech) supplemented with 0.4 µM PD0325901, 1 µM CHIR99021, 5 µM Y-27632 and 2 µM SB431542. To generate stable iPS cell lines with integrated Dox-inducible *MYOD1* transgene, iPS cells were infected with the MyoD lentivirus supplemented with 4 µg ml^−1^ polybrene (TR-1003-G, Sigma). Uninfected cells were removed by 3-day incubation with 2 µg ml^−1^ puromycin (AT1113803, Gibco) to obtain a pure population of transduced cells. Following selection, iPS cells were pooled and expanded in growth media with puromycin. To prime cells for differentiation, iPS cells were seeded on Matrigel-coated plates in iPS cell growth media without bFGF supplemented with ROCK inhibitor (Y-27632). The next day, the medium was changed to induction medium (DMEM and 15% FBS) containing 3 µg ml^−1^ doxycycline (D9891, Sigma) to induce MyoD transgene expression. Medium was changed 4 days later to Dox-containing differentiation media (low-glucose DMEM (36253, StemCell Tech), 5% horse serum (26050088, Thermo Fisher) and 3 µg ml^−1^ doxycycline) and changed every 2 days.

### Cerebral organoid differentiation

Cerebral organoid differentiation was based on previous established protocols with slight modifications^48^. In brief, iPS cells were dissociated and centrifuged at 300*g* for 3 min. Cells were resuspended in mTeSR1 media plus 10 μM ROCK inhibitor and single celled by gentle pipetting. Ten thousand cells were plated in a low-attachment 96-well plate, followed by centrifugation at 100*g* for 3 min and placed in an incubator at 37 °C at 5% CO_2_. Medium was changed every other day for 6–7 days. On days 6–7, when embryoid bodies have smooth edges and begin to brighten, the medium was changed to neural induction media (DMEM/F12, 1:100 N2 supplement (17502048, Invitrogen), Glutamax, MEM-NEAA and 1 µg ml^−1^ heparin (H3149, Sigma)) and refed every other day. After 4–5 days, neuroepithelia became visible and aggregates were coated in a 1% diluted Matrigel–DMEM solution and incubated at 37 °C for at least 30 min to allow Matrigel polymerization. Differentiation media without vitamin A (1:1 mixture of DMEM/F12 and neurobasal (21103049, Invitrogen) containing 1:200 N2 supplement, 1:100 B27 supplement without vitamin A (12587010, Invitrogen), 3.5 µl l^−1^ 2-mercaptoethanol, 1:4,000 insulin (I9278, Sigma), 1:100 Glutamax and 1:200 MEM-NEAA) were added and refed every other day. After 3–4 days, differentiation media were changed to differentiation media with vitamin A (17504044, Invitrogen) and changed every 3–4 days. Cerebral organoids were ready for downstream analysis after 2–3 weeks.

### Ethics statement

The Institutional Review Board of Children’s Mercy Research Institute gave ethical approval for this work (studies #00002465 and #11120514). All methods were carried out in accordance with relevant guidelines and regulations.

### Reporting summary

Further information on research design is available in the Nature Portfolio Reporting Summary linked to this article.

## Online content

Any methods, additional references, Nature Portfolio reporting summaries, source data, extended data, supplementary information, acknowledgements, peer review information; details of author contributions and competing interests; and statements of data and code availability are available at 10.1038/s41586-024-08462-1.

## Supplementary information

Supplementary Figures Supplementary Figs. 1–13

Reporting Summary

Supplementary Table 1 Summary reprogramming reagents and methods

Supplementary Table 2 Summary of iPSC lines.

Supplementary Table 3 **ASO sequences and targets**. All ASOs in this study were synthesized as fully 2’-O-methyl-modified RNAs.

Supplementary Table 4 Primary antibodies used in this study

Supplementary Table 5 PCR primers used in this study.

Supplementary Videos Supplementary Videos 1–47

## Data Availability

The BioProject accession number for the amplicon sequencing data described in this study is PRJNA1177506. Genome sequencing data collected through GA4K are de-identified and have been deposited in dbGaP (accession: phs002206.v2.p1).
