# Supplementary material for: Rapid and scalable personalized ASO screening in patient-derived organoids
Source: Nature. 2025 Jan 22;638(8049):237–43. doi: 10.1038/s41586-024-08462-1 (PMC11798851; doi:10.1038/s41586-024-08462-1)
Supplement: Supplementary file 2 — Reporting Summary [file 41586_2024_8462_MOESM2_ESM.pdf]

nature portfolio

Corresponding author(s): Scott Younger

Last updated by author(s): Aug 28, 2024

Reporting Summary

Nature Portfolio wishes to improve the reproducibility of the work that we publish. This form provides structure for consistency and transparency in reporting. For further information on Nature Portfolio policies, see our Editorial Policies and the Editorial Policy Checklist.

Statistics

For all statistical analyses, confirm that the following items are present in the figure legend, table legend, main text, or Methods section.

n/a	Confirmed	
☐	☒	The exact sample size (*n*) for each experimental group/condition, given as a discrete number and unit of measurement
☐	☒	A statement on whether measurements were taken from distinct samples or whether the same sample was measured repeatedly
☒	☐	The statistical test(s) used AND whether they are one- or two-sided *Only common tests should be described solely by name; describe more complex techniques in the Methods section.*
☒	☐	A description of all covariates tested
☒	☐	A description of any assumptions or corrections, such as tests of normality and adjustment for multiple comparisons
☐	☒	A full description of the statistical parameters including central tendency (e.g. means) or other basic estimates (e.g. regression coefficient) AND variation (e.g. standard deviation) or associated estimates of uncertainty (e.g. confidence intervals)
☒	☐	For null hypothesis testing, the test statistic (e.g. *F*, *t*, *r*) with confidence intervals, effect sizes, degrees of freedom and *P* value noted *Give P values as exact values whenever suitable.*
☒	☐	For Bayesian analysis, information on the choice of priors and Markov chain Monte Carlo settings
☒	☐	For hierarchical and complex designs, identification of the appropriate level for tests and full reporting of outcomes
☒	☐	Estimates of effect sizes (e.g. Cohen's *d*, Pearson's *r*), indicating how they were calculated

Our web collection on statistics for biologists contains articles on many of the points above.

Software and code

Policy information about availability of computer code

Data collection	FlowJo (version 10)
Data analysis	ImageJ (version 1.53k)

For manuscripts utilizing custom algorithms or software that are central to the research but not yet described in published literature, software must be made available to editors and reviewers. We strongly encourage code deposition in a community repository (e.g. GitHub). See the Nature Portfolio guidelines for submitting code & software for further information.

Data

Policy information about availability of data

All manuscripts must include a data availability statement. This statement should provide the following information, where applicable:

- Accession codes, unique identifiers, or web links for publicly available datasets
- A description of any restrictions on data availability
- For clinical datasets or third party data, please ensure that the statement adheres to our policy

All data are available upon request from the corresponding author.

Research involving human participants, their data, or biological material

Policy information about studies with human participants or human data. See also policy information about sex, gender (identity/presentation), and sexual orientation and race, ethnicity and racism.

Reporting on sex and gender	Females: 7785, Males: 6068
Reporting on race, ethnicity, or other socially relevant groupings	American Indian/Alaskan Native: 59, Asian: 180, Native Hawaiian or Other Pacific Islander: 30, Black or African American: 552, White: 11090, More than One Race: 558, Unknown or Not Reported: 1384
Population characteristics	Pediatric Rare disease
Recruitment	Potential participants are informed of our GA4K program by clinicians in our health system. Alternatively, participants may hear of the GA4K program by word of mouth.
Ethics oversight	Institutional Review Board (IRB) of Children's Mercy Research Institute

Note that full information on the approval of the study protocol must also be provided in the manuscript.

Field-specific reporting

Please select the one below that is the best fit for your research. If you are not sure, read the appropriate sections before making your selection.

☒ Life sciences ☐ Behavioural & social sciences ☐ Ecological, evolutionary & environmental sciences

For a reference copy of the document with all sections, see nature.com/documents/nr-reporting-summary-flat.pdf

Life sciences study design

All studies must disclose on these points even when the disclosure is negative.

Sample size	This study did not include experiments that require sample size calculations. Experiments were focused on participants in a rare disease cohort with genetic variants that are amenable to treatment with ASO therapeutics.
Data exclusions	No exclusions
Replication	All experiments were repeated in at least 3 biological replicates. All attempts at replication were successful.
Randomization	This study did not include experiments that require sample randomization
Blinding	This study did not include experiments that require blinding.

Reporting for specific materials, systems and methods

We require information from authors about some types of materials, experimental systems and methods used in many studies. Here, indicate whether each material, system or method listed is relevant to your study. If you are not sure if a list item applies to your research, read the appropriate section before selecting a response.

Materials & experimental systems

n/a	Involved in the study	
☐	☒	Antibodies
☐	☒	Eukaryotic cell lines
☒	☐	Palaeontology and archaeology
☒	☐	Animals and other organisms
☒	☐	Clinical data
☒	☐	Dual use research of concern
☒	☐	Plants

Methods

n/a	Involved in the study	
☒	☐	ChIP-seq
☐	☒	Flow cytometry
☒	☐	MRI-based neuroimaging

Antibodies

Antibodies used	OCT4, Cell Signaling, #2750<br>SOX2, Cell Signaling, #3579<br>NANOG, Cell Signaling, #3580

TRA-1-60, Cell Signaling, #4746
TRA-1-81, Cell Signaling, #4745
SSEA4, Cell Signaling, #4755
BRACHYURY, Cell Signaling, #81694
FOXA2, Cell Signaling, #8186
SOX17, Cell Signaling, #81778
PAX6, Cell Signaling, #60433
NCAM (CD56), StemCell Tech, #60021
NESTIN, StemCell tech, #60091
FOXG1, Thermofisher, PA5-26794
NKX2.5, Thermofisher, PA5-49431
Myosin Heavy Chain (MHC), Thermofisher, MA5-35613
HAND1, Thermofisher, MA5-25494
CD31, Thermofisher, MA5-13188
Cardiac Troponin T (TNNT2), Thermofisher, MA5-12960
Beta-3 Tubulin (TUJI), Thermofisher, MA1-118
Dystrophin, Abcam, ab15277
Viniculin, Invitrogen, 14-97777-82
Anti-Rabbit Alexa Fluor 488 Goat Fisher, A11034 1:400
Anti-Mouse Alexa Fluor 594 Goat Fisher, A11032 1:400
Anti-Mouse IRDye800CW Goat LI-COR, 926-32210 1:5000
Anti-Rabbit IRDye800CW Goat LI-COR, 926-32211 1:5000

Validation

All antibodies were validated by vendors. OCT4, NANOG were validated by chromatin IP. CD31, Viniculin were validated by knockout. TNNT2, TUJI were validated by cell treatment. All other antibodies were validated by relative expression.

Eukaryotic cell lines

Policy information about cell lines and Sex and Gender in Research

Cell line source(s)

Control human iPSC line (WTC11 background) was obtained from Coriell Institute (GM25256), HEK293T cells were obtained from ATCC, Induced pluripotent stem cells derived from patients

Authentication

Karyotype, targeted genotyping

Mycoplasma contamination

Cells not tested for mycoplasma contamination

Commonly misidentified lines
(See ICLAC register)

No commonly misidentified cell lines were used in this study

Plants

Seed stocks

Report on the source of all seed stocks or other plant material used. If applicable, state the seed stock centre and catalogue number. If plant specimens were collected from the field, describe the collection location, date and sampling procedures.

Novel plant genotypes

Describe the methods by which all novel plant genotypes were produced. This includes those generated by transgenic approaches, gene editing, chemical/radiation-based mutagenesis and hybridization. For transgenic lines, describe the transformation method, the number of independent lines analyzed and the generation upon which experiments were performed. For gene-edited lines, describe the editor used, the endogenous sequence targeted for editing, the targeting guide RNA sequence (if applicable) and how the editor was applied.

Authentication

Describe any authentication procedures for each seed stock used or novel genotype generated. Describe any experiments used to assess the effect of a mutation and, where applicable, how potential secondary effects (e.g. second site T-DNA insertions, mosiacism, off-target gene editing) were examined.

Flow Cytometry

Plots

Confirm that:

☒ The axis labels state the marker and fluorochrome used (e.g. CD4-FITC).

☒ The axis scales are clearly visible. Include numbers along axes only for bottom left plot of group (a 'group' is an analysis of identical markers).

☒ All plots are contour plots with outliers or pseudocolor plots.

☒ A numerical value for number of cells or percentage (with statistics) is provided.

Methodology

Sample preparation

Three days after organoid transfection organoids were dissociated using TrypLE Express (Thermofisher, 126040132). Organoids were washed once with 1xPBS and then incubated in TrypLE solution at 37 °C for 30 min. Subsequently, organoids

were dissociated into single cells by gentle pipetting. Cells were then centrifuged down to remove TrypLE and washed once with 1xPBS (300×g for 3 min). Pellets were resuspended in 1XPBS supplemented with 2% FBS and 4mM EDTA in a final volume of 300 uL and stained with 7AAD for 15 min at 4°C in the dark. Cells were washed twice with PBS/FBS/EDTA then analyzed

Instrument	BD Fortessa X-20
Software	FlowJo v10
Cell population abundance	The fraction of ASO-containing cells was measured by gating on total nucleated cells (TNC; FSC/SSC), live cells (7AAD negative), then percentage of fluorescence positive cells in the FITC channel. An untransfected control was used to subtract baseline fluorescence intensity.
Gating strategy	The fraction of ASO-containing cells was measured by gating on total nucleated cells (TNC; FSC/SSC), live cells (7AAD negative), then percentage of fluorescence positive cells in the FITC channel. An untransfected control was used to subtract baseline fluorescence intensity.

☒ Tick this box to confirm that a figure exemplifying the gating strategy is provided in the Supplementary Information.
