# Supplementary material for: Rapid and scalable personalized ASO screening in patient-derived organoids
Source: Nature. 2025 Jan 22;638(8049):237–43. doi: 10.1038/s41586-024-08462-1 (PMC11798851; doi:10.1038/s41586-024-08462-1)
Supplement: Supplementary file 8 — Supplementary Videos 1–47 [file 41586_2024_8462_MOESM8_ESM.zip › 2023-03-05322C-s8/Supplementary Video Legends.docx]

**Supplementary Video Legends**

**Supplementary Video 1. Fluo-4 imaging of a cardiac organoid.** Cardiac organoid generated from a commercially available iPSC line.

**Supplementary Video 2. Fluo-4 imaging of a cardiac organoid.** Cardiac organoid generated from a commercially available iPSC line and treated with a nontargeting ASO post-differentiation.

**Supplementary Video 3. Fluo-4 imaging of a cardiac organoid.** Cardiac organoid generated from a commercially available iPSC line and treated with a scrambled ASO (based on cardiac troponin T AUG-targeting ASO) post-differentiation.

**Supplementary Video 4. Fluo-4 imaging of a cardiac organoid.** Cardiac organoid generated from a commercially available iPSC line and treated with a mismatched ASO (based on cardiac troponin T AUG-targeting ASO) post-differentiation.

**Supplementary Video 5. Fluo-4 imaging of a cardiac organoid.** Cardiac organoid generated from a commercially available iPSC line and treated with an ASO targeting the cardiac troponin T AUG translation start site post-differentiation.

**Supplementary Video 6. Fluo-4 imaging of a cardiac organoid.** Cardiac organoid generated from a commercially available iPSC line and treated with an ASO targeting a splice donor site in the cardiac troponin T gene post-differentiation.

**Supplementary Video 7. Fluo-4 imaging of a cardiac organoid.** Cardiac organoid generated from a commercially available iPSC line and treated with an ASO targeting a splice acceptor site in the cardiac troponin T gene post-differentiation.

**Supplementary Video 8. Fluo-4 imaging of a cardiac organoid.** Cardiac organoid generated from a commercially available iPSC line.

**Supplementary Video 9. Fluo-4 imaging of a cardiac organoid.** Cardiac organoid generated from an iPSC line derived from Patient 1.

**Supplementary Video 10. Fluo-4 imaging of a cardiac organoid.** Cardiac organoid generated from an iPSC line derived from Patient 1 and treated with a nontargeting ASO.

**Supplementary Video 11. Fluo-4 imaging of a cardiac organoid.** Cardiac organoid generated from an iPSC line derived from Patient 1 and treated with a DMD exon 45 skipping ASO matching the sequence of an FDA-approved ASO.

**Supplementary Video 12. Fluo-4 imaging of a cardiac organoid.** Cardiac organoid generated from an iPSC line derived from Patient 1 and treated with a DMD exon 45 skipping ASO matching the sequence of an ASO in phase II clinical trials.

**Supplementary Video 13. Fluo-4 imaging of a cardiac organoid.** Cardiac organoid generated from a commercially available iPSC line using an alternative differentiation protocol.

**Supplementary Video 14. Fluo-4 imaging of a cardiac organoid.** Cardiac organoid generated from an iPSC line derived from Patient 1 using an alternative differentiation protocol.

**Supplementary Video 15. Fluo-4 imaging of a cardiac organoid.** Cardiac organoid generated from an iPSC line derived from Patient 1 and treated with a nontargeting ASO using an alternative differentiation protocol.

**Supplementary Video 16. Fluo-4 imaging of a cardiac organoid.** Cardiac organoid generated from an iPSC line derived from Patient 1 and treated with a DMD exon 45 skipping ASO matching the sequence of an FDA-approved ASO using an alternative differentiation protocol.

**Supplementary Video 17. Fluo-4 imaging of a cardiac organoid.** Cardiac organoid generated from an iPSC line derived from Patient 1 and treated with a DMD exon 45 skipping ASO matching the sequence of an ASO in phase II clinical trials using an alternative differentiation protocol.

**Supplementary Video 18. Fluo-4 imaging of a cardiac organoid.** Cardiac organoid generated from a commercially available iPSC line.

**Supplementary Video 19. Fluo-4 imaging of a cardiac organoid.** Cardiac organoid generated from an iPSC line derived from Patient 2a.

**Supplementary Video 20. Fluo-4 imaging of a cardiac organoid.** Cardiac organoid generated from an iPSC line derived from Patient 2a and treated with a mismatched ASO (based on ASO-1).

**Supplementary Video 21. Fluo-4 imaging of a cardiac organoid.** Cardiac organoid generated from an iPSC line derived from Patient 2a and treated with a scrambled ASO (based on ASO-1).

**Supplementary Video 22. Fluo-4 imaging of a cardiac organoid.** Cardiac organoid generated from an iPSC line derived from Patient 2a and treated with ASO-1.

**Supplementary Video 23. Fluo-4 imaging of a cardiac organoid.** Cardiac organoid generated from an iPSC line derived from Patient 2a and treated with a mismatched ASO (based on ASO-2).

**Supplementary Video 24. Fluo-4 imaging of a cardiac organoid.** Cardiac organoid generated from an iPSC line derived from Patient 2a and treated with a scrambled ASO (based on ASO-2).

**Supplementary Video 25. Fluo-4 imaging of a cardiac organoid.** Cardiac organoid generated from an iPSC line derived from Patient 2a and treated with ASO-2.

**Supplementary Video 26. Fluo-4 imaging of a cardiac organoid.** Cardiac organoid generated from an iPSC line derived from Patient 2a using an alternative differentiation protocol.

**Supplementary Video 27. Fluo-4 imaging of a cardiac organoid.** Cardiac organoid generated from an iPSC line derived from Patient 2a using an alternative differentiation protocol and treated with a mismatched ASO (based on ASO-1).

**Supplementary Video 28. Fluo-4 imaging of a cardiac organoid.** Cardiac organoid generated from an iPSC line derived from Patient 2a using an alternative differentiation protocol and treated with a scrambled ASO (based on ASO-1).

**Supplementary Video 29. Fluo-4 imaging of a cardiac organoid.** Cardiac organoid generated from an iPSC line derived from Patient 2a using an alternative differentiation protocol and treated with ASO-1.

**Supplementary Video 30. Fluo-4 imaging of a cardiac organoid.** Cardiac organoid generated from an iPSC line derived from Patient 2a using an alternative differentiation protocol and treated with a mismatched ASO (based on ASO-2).

**Supplementary Video 31. Fluo-4 imaging of a cardiac organoid.** Cardiac organoid generated from an iPSC line derived from Patient 2a using an alternative differentiation protocol and treated with a scrambled ASO (based on ASO-2).

**Supplementary Video 32. Fluo-4 imaging of a cardiac organoid.** Cardiac organoid generated from an iPSC line derived from Patient 2a using an alternative differentiation protocol and treated with ASO-2.

**Supplementary Video 33. Fluo-4 imaging of a cardiac organoid.** Cardiac organoid generated from a commercially available iPSC line.

**Supplementary Video 34. Fluo-4 imaging of a cardiac organoid.** Cardiac organoid generated from an iPSC line derived from Patient 2b.

**Supplementary Video 35. Fluo-4 imaging of a cardiac organoid.** Cardiac organoid generated from an iPSC line derived from Patient 2b and treated with a mismatched ASO (based on ASO-1).

**Supplementary Video 36. Fluo-4 imaging of a cardiac organoid.** Cardiac organoid generated from an iPSC line derived from Patient 2b and treated with a scrambled ASO (based on ASO-1).

**Supplementary Video 37. Fluo-4 imaging of a cardiac organoid.** Cardiac organoid generated from an iPSC line derived from Patient 2b and treated with ASO-1.

**Supplementary Video 38. Fluo-4 imaging of a cardiac organoid.** Cardiac organoid generated from an iPSC line derived from Patient 2b and treated with a mismatched ASO (based on ASO-2).

**Supplementary Video 39. Fluo-4 imaging of a cardiac organoid.** Cardiac organoid generated from an iPSC line derived from Patient 2b and treated with a scrambled ASO (based on ASO-2).

**Supplementary Video 40. Fluo-4 imaging of a cardiac organoid.** Cardiac organoid generated from an iPSC line derived from Patient 2b and treated with ASO-2.

**Supplementary Video 41. Fluo-4 imaging of a cardiac organoid.** Cardiac organoid generated from an iPSC line derived from Patient 2b using an alternative differentiation protocol.

**Supplementary Video 42. Fluo-4 imaging of a cardiac organoid.** Cardiac organoid generated from an iPSC line derived from Patient 2b using an alternative differentiation protocol and treated with a mismatched ASO (based on ASO-1).

**Supplementary Video 43. Fluo-4 imaging of a cardiac organoid.** Cardiac organoid generated from an iPSC line derived from Patient 2b using an alternative differentiation protocol and treated with a scrambled ASO (based on ASO-1).

**Supplementary Video 44. Fluo-4 imaging of a cardiac organoid.** Cardiac organoid generated from an iPSC line derived from Patient 2b using an alternative differentiation protocol and treated with ASO-1.

**Supplementary Video 45. Fluo-4 imaging of a cardiac organoid.** Cardiac organoid generated from an iPSC line derived from Patient 2b using an alternative differentiation protocol and treated with a mismatched ASO (based on ASO-2).

**Supplementary Video 46. Fluo-4 imaging of a cardiac organoid.** Cardiac organoid generated from an iPSC line derived from Patient 2b using an alternative differentiation protocol and treated with a scrambled ASO (based on ASO-2).

**Supplementary Video 47. Fluo-4 imaging of a cardiac organoid.** Cardiac organoid generated from an iPSC line derived from Patient 2b using an alternative differentiation protocol and treated with ASO-2.
