# Supplementary material for: Rapid and scalable personalized ASO screening in patient-derived organoids
Source: Nature. 2025 Jan 22;638(8049):237–43. doi: 10.1038/s41586-024-08462-1 (PMC11798851; doi:10.1038/s41586-024-08462-1)
Supplement: Supplementary file 1 — Supplementary Figs. 1–13 [file 41586_2024_8462_MOESM1_ESM.zip › 2023-03-05322C-s1/SupplementaryFigure11.pdf]

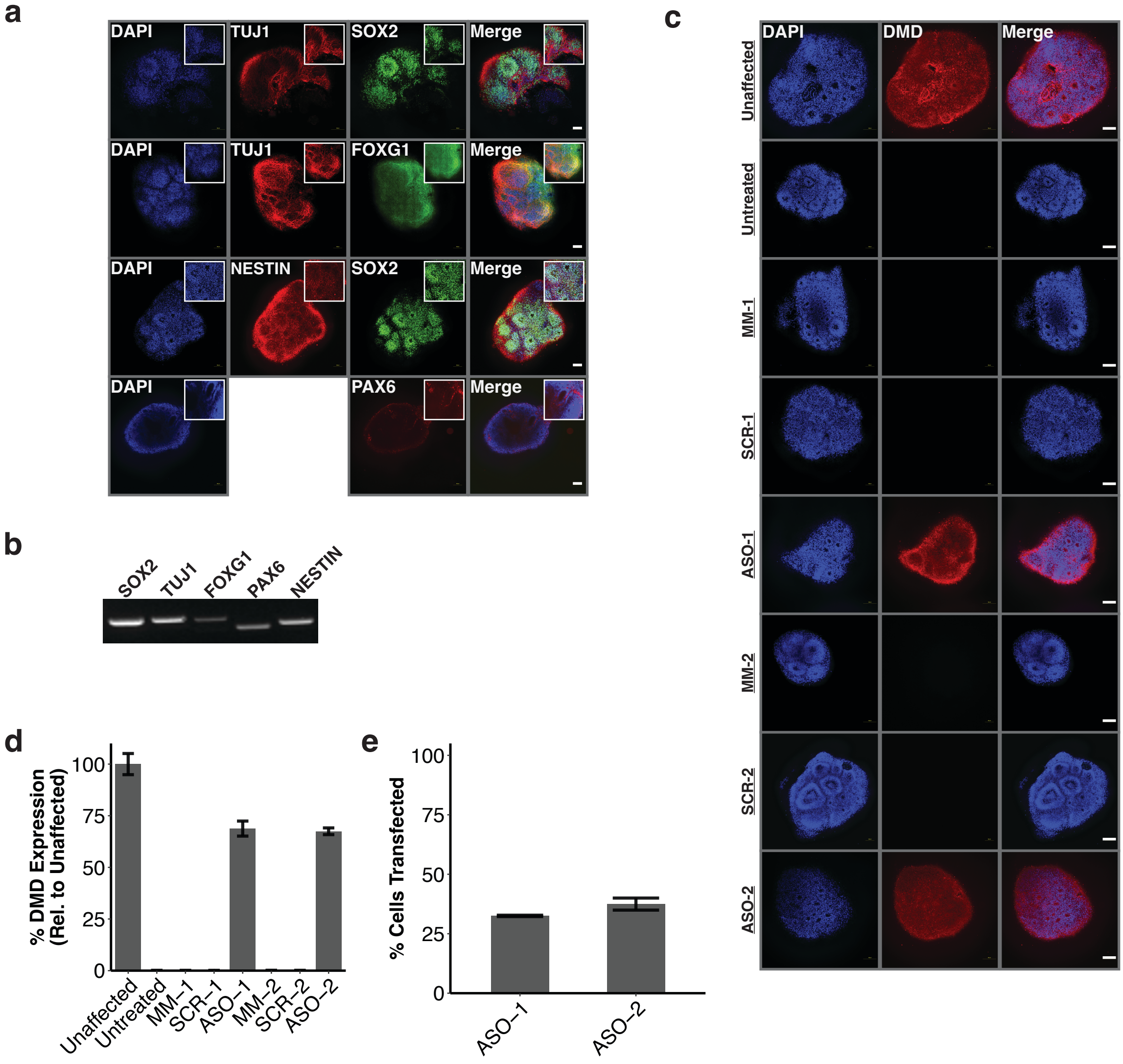
a
DAPI
TUJ1
SOX2
Merge
FOXG1
NESTIN
PAX6
b
SOX2
TUJ1
FOXG1
PAX6
NESTIN
c
DAPI
DMD
Merge
Unaffected
Untreated
MM-1
SCR-1
ASO-1
MM-2
SCR-2
ASO-2
d
% DMD Expression
(Rel. to Unaffected)
0
25
50
75
100
Unaffected
Untreated
MM–1
SCR–1
ASO–1
MM–2
SCR–2
ASO–2
e
% Cells Transfected
0
25
50
75
100
ASO–1
ASO–2
