# Supplementary material for: Rapid and scalable personalized ASO screening in patient-derived organoids
Source: Nature. 2025 Jan 22;638(8049):237–43. doi: 10.1038/s41586-024-08462-1 (PMC11798851; doi:10.1038/s41586-024-08462-1)
Supplement: Supplementary file 1 — Supplementary Figs. 1–13 [file 41586_2024_8462_MOESM1_ESM.zip › 2023-03-05322C-s1/SupplementaryFigure12.pdf]

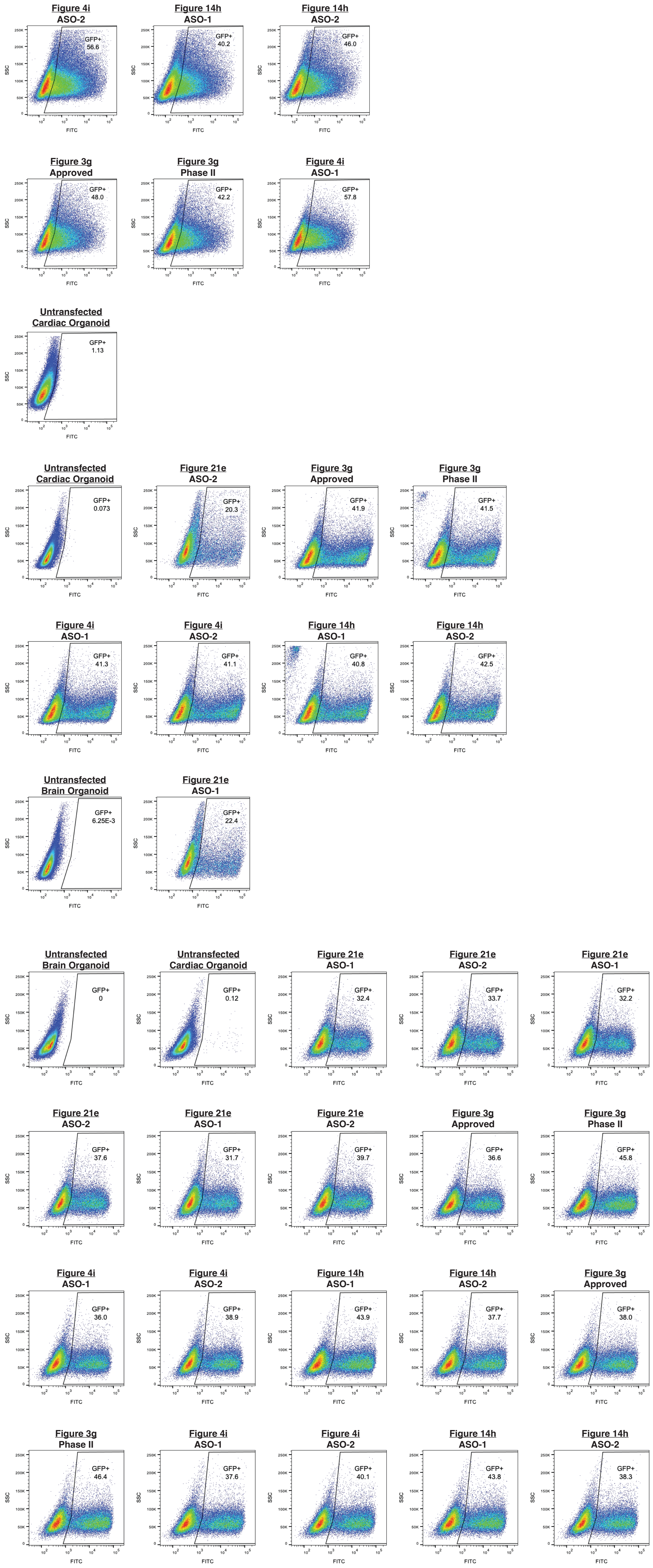
Figure 4i ASO-2
GFP+ 56.6
Figure 14h ASO-1
GFP+ 40.2
Figure 14h ASO-2
GFP+ 46.0
Figure 3g Approved
GFP+ 48.0
Figure 3g Phase II
GFP+ 42.2
Figure 4i ASO-1
GFP+ 57.8
Untransfected Cardiac Organoid
GFP+ 1.13
Untransfected Cardiac Organoid
GFP+ 0.073
Figure 21e ASO-2
GFP+ 20.3
Figure 3g Approved
GFP+ 41.9
Figure 3g Phase II
GFP+ 41.5
Figure 4i ASO-1
GFP+ 41.3
Figure 4i ASO-2
GFP+ 41.1
Figure 14h ASO-1
GFP+ 40.8
Figure 14h ASO-2
GFP+ 42.5
Untransfected Brain Organoid
GFP+ 6.25E-3
Figure 21e ASO-1
GFP+ 22.4
Untransfected Brain Organoid
GFP+ 0
Untransfected Cardiac Organoid
GFP+ 0.12
Figure 21e ASO-1
GFP+ 32.4
Figure 21e ASO-2
GFP+ 33.7
Figure 21e ASO-1
GFP+ 32.2
Figure 21e ASO-2
GFP+ 37.6
Figure 21e ASO-1
GFP+ 31.7
Figure 21e ASO-2
GFP+ 39.7
Figure 3g Approved
GFP+ 36.6
Figure 3g Phase II
GFP+ 45.8
Figure 4i ASO-1
GFP+ 36.0
Figure 4i ASO-2
GFP+ 38.9
Figure 14h ASO-1
GFP+ 43.9
Figure 14h ASO-2
GFP+ 37.7
Figure 3g Approved
GFP+ 38.0
Figure 3g Phase II
GFP+ 46.4
Figure 4i ASO-1
GFP+ 37.6
Figure 4i ASO-2
GFP+ 40.1
Figure 14h ASO-1
GFP+ 43.8
Figure 14h ASO-2
GFP+ 38.3
