# Supplementary material for: Rapid and scalable personalized ASO screening in patient-derived organoids
Source: Nature. 2025 Jan 22;638(8049):237–43. doi: 10.1038/s41586-024-08462-1 (PMC11798851; doi:10.1038/s41586-024-08462-1)
Supplement: Supplementary file 1 — Supplementary Figs. 1–13 [file 41586_2024_8462_MOESM1_ESM.zip › 2023-03-05322C-s1/SupplementaryFigure13.pdf]

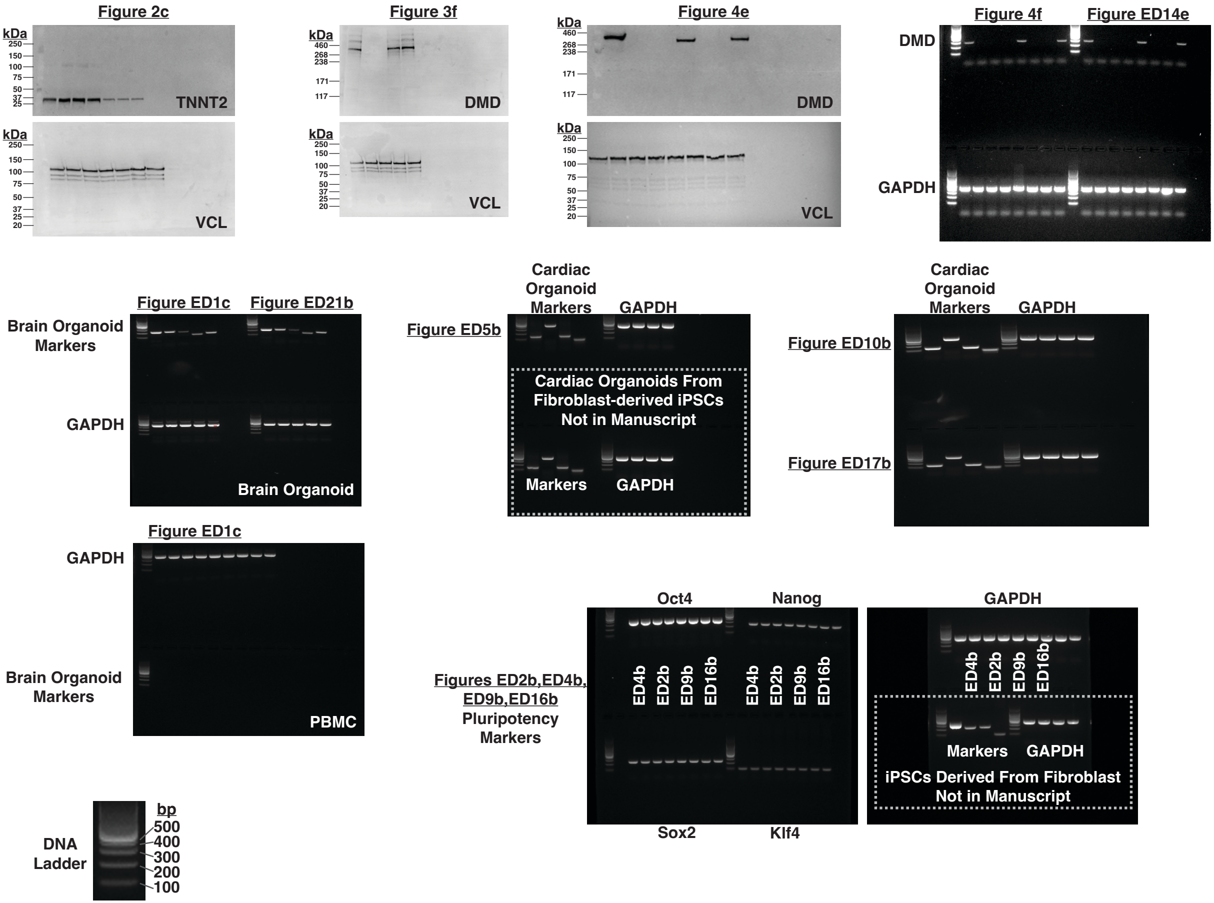
Figure 2c
kDa
250
150
100
75
50
37
25
TNNT2
kDa
250
150
100
75
50
37
25
20
VCL
Figure 3f
kDa
460
268
238
171
117
DMD
kDa
250
150
100
75
50
37
25
20
VCL
Figure 4e
kDa
460
268
238
171
117
DMD
kDa
250
150
100
75
50
37
25
20
VCL
Figure 4f
Figure ED14e
DMD
GAPDH
Figure ED1c
Figure ED21b
Brain Organoid Markers
GAPDH
Brain Organoid
Cardiac Organoid Markers
GAPDH
Figure ED5b
Cardiac Organoids From Fibroblast-derived iPSCs Not in Manuscript
Markers
GAPDH
Cardiac Organoid Markers
GAPDH
Figure ED10b
Figure ED17b
Figure ED1c
GAPDH
Brain Organoid Markers
PBMC
Oct4
Nanog
GAPDH
Figures ED2b,ED4b, ED9b,ED16b Pluripotency Markers
ED4b
ED2b
ED9b
ED16b
ED4b
ED2b
ED9b
ED16b
ED4b
ED2b
ED9b
ED16b
Markers
GAPDH
iPSCs Derived From Fibroblast Not in Manuscript
Sox2
Klf4
DNA Ladder
bp
500
400
300
200
100
