# Supplementary material for: Rapid and scalable personalized ASO screening in patient-derived organoids
Source: Nature. 2025 Jan 22;638(8049):237–43. doi: 10.1038/s41586-024-08462-1 (PMC11798851; doi:10.1038/s41586-024-08462-1)
Supplement: Supplementary file 1 — Supplementary Figs. 1–13 [file 41586_2024_8462_MOESM1_ESM.zip › 2023-03-05322C-s1/SupplementaryFigure2.pdf]

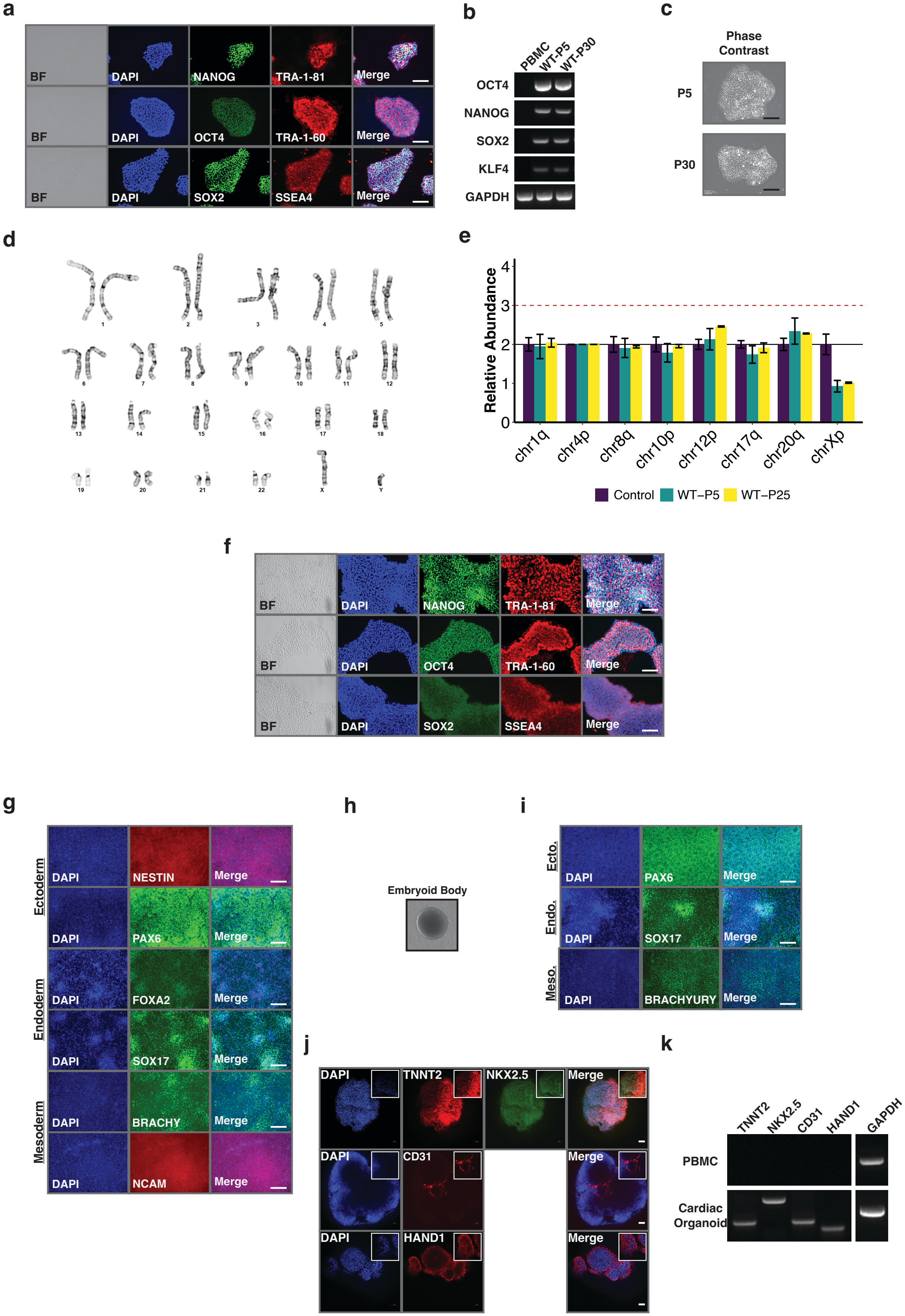
a
BF
DAPI
NANOG
TRA-1-81
Merge
OCT4
TRA-1-60
SOX2
SSEA4
b
PBMC
WT-P5
WT-P30
OCT4
NANOG
SOX2
KLF4
GAPDH
c
Phase Contrast
P5
P30
d
e
Relative Abundance
0
1
2
3
4
chr1q
chr4p
chr8q
chr10p
chr12p
chr17q
chr20q
chrXp
Control
WT–P5
WT–P25
f
BF
DAPI
NANOG
TRA-1-81
Merge
OCT4
TRA-1-60
SOX2
SSEA4
g
Ectoderm
Endoderm
Mesoderm
DAPI
NESTIN
PAX6
FOXA2
SOX17
BRACHY
NCAM
Merge
h
Embryoid Body
i
Ecto.
Endo.
Meso.
DAPI
PAX6
SOX17
BRACHYURY
Merge
j
DAPI
TNNT2
NKX2.5
CD31
HAND1
Merge
k
TNNT2
NKX2.5
CD31
HAND1
GAPDH
PBMC
Cardiac Organoid
