# Supplementary material for: Rapid and scalable personalized ASO screening in patient-derived organoids
Source: Nature. 2025 Jan 22;638(8049):237–43. doi: 10.1038/s41586-024-08462-1 (PMC11798851; doi:10.1038/s41586-024-08462-1)
Supplement: Supplementary file 1 — Supplementary Figs. 1–13 [file 41586_2024_8462_MOESM1_ESM.zip › 2023-03-05322C-s1/SupplementaryFigure4.pdf]

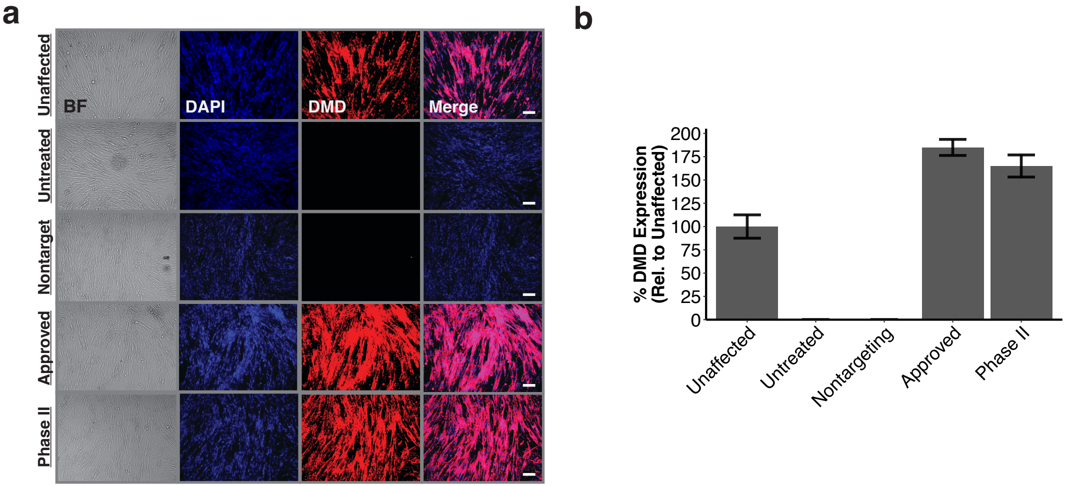
a
Unaffected
Untreated
Nontarget
Approved
Phase II
BF
DAPI
DMD
Merge
b
% DMD Expression
(Rel. to Unaffected)
200
175
150
125
100
75
50
25
0
Unaffected
Untreated
Nontargeting
Approved
Phase II
