# Supplementary material for: Rapid and scalable personalized ASO screening in patient-derived organoids
Source: Nature. 2025 Jan 22;638(8049):237–43. doi: 10.1038/s41586-024-08462-1 (PMC11798851; doi:10.1038/s41586-024-08462-1)
Supplement: Supplementary file 1 — Supplementary Figs. 1–13 [file 41586_2024_8462_MOESM1_ESM.zip › 2023-03-05322C-s1/SupplementaryFigure6.pdf]

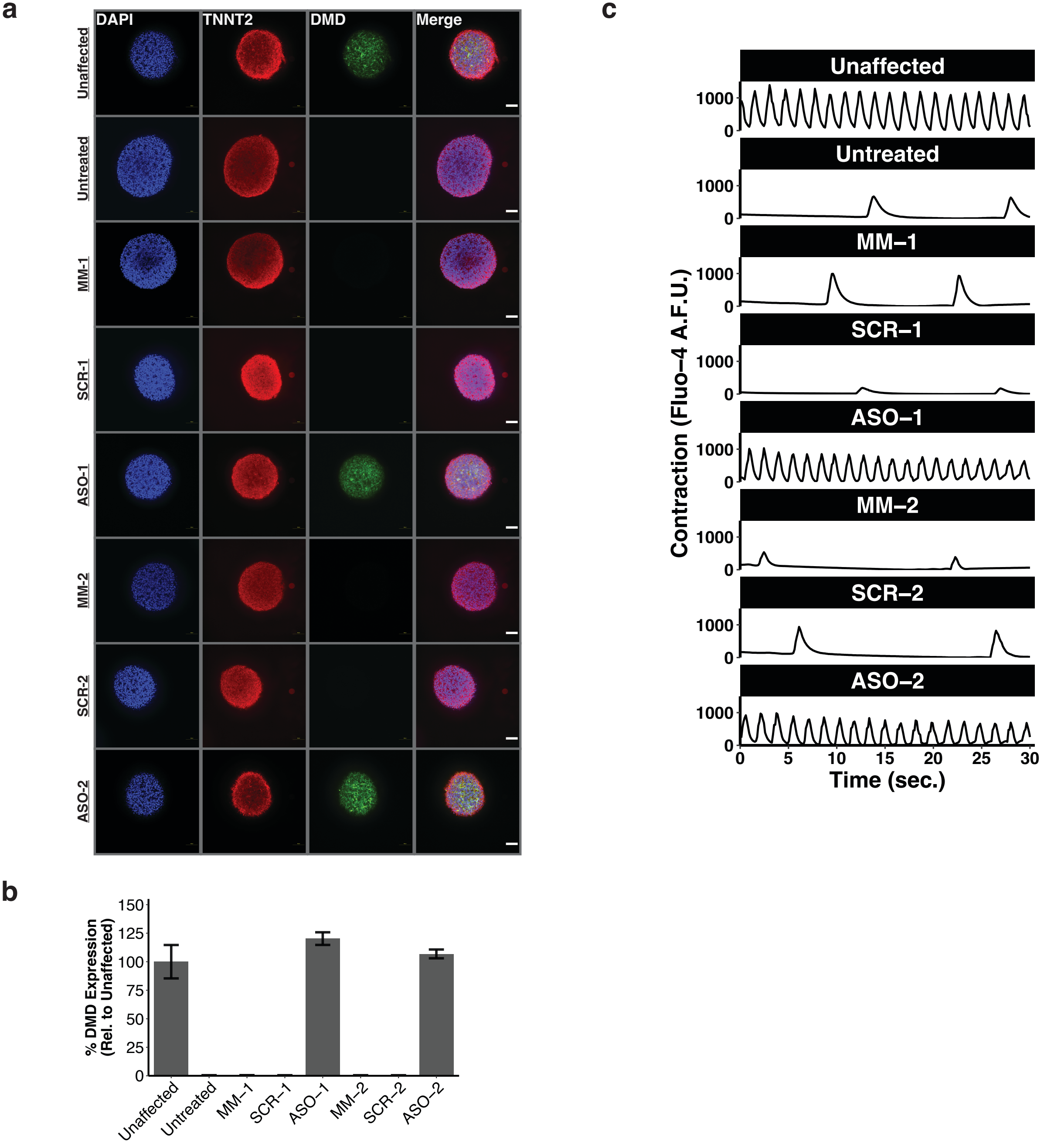
a
DAPI
TNNT2
DMD
Merge
Unaffected
Untreated
MM-1
SCR-1
ASO-1
MM-2
SCR-2
ASO-2
b
% DMD Expression (Rel. to Unaffected)
150
125
100
75
50
25
0
Unaffected
Untreated
MM–1
SCR–1
ASO–1
MM–2
SCR–2
ASO–2
c
Unaffected
Untreated
MM–1
SCR–1
ASO–1
MM–2
SCR–2
ASO–2
1000
0
Contraction (Fluo–4 A.F.U.)
0
5
10
15
20
25
30
Time (sec.)
