# Supplementary material for: Rapid and scalable personalized ASO screening in patient-derived organoids
Source: Nature. 2025 Jan 22;638(8049):237–43. doi: 10.1038/s41586-024-08462-1 (PMC11798851; doi:10.1038/s41586-024-08462-1)
Supplement: Supplementary file 1 — Supplementary Figs. 1–13 [file 41586_2024_8462_MOESM1_ESM.zip › 2023-03-05322C-s1/SupplementaryFigureLegends.docx]

**Supplementary Figure Legends**

**Supplementary Figure 1. Patient-derived iPSCs remain stable over extended culturing. a)** iPSC marker expression in patient-derived iPSCs (patient 1) after 30 passages. **b)** iPSC marker expression in patient-derived iPSCs (patient 1) after 5 and 30 passages. **c)** Phase contrast imaging of patient-derived iPSC colonies (patient 1) after 5 and 30 passages. **d)** qPCR analysis of common karyotypic abnormalities in patient-derived iPSCs (patient 1) after 5 and 25 passages. **e)** Differentiation of late-passage (p30) patient-derived iPSCs (patient 1) into ectoderm, endoderm, and mesoderm lineages. Data in **b** are representative of *n* = 3 biologically independent experiments. Error bars in **d** represent standard deviation for *n* = 3 replicates. Scale bars in **a, c, e** represent 100um.

**Supplementary Figure 2. Characterization of commercially available iPSCs. a)** iPSC marker expression in iPSCs after 5 passages. **b)** iPSC marker expression in iPSCs after 5 and 30 passages. **c)** Phase contrast imaging of iPSC colonies after 5 and 30 passages. **d)** Representative karyotype of iPSCs. **e)** qPCR analysis of common karyotypic abnormalities in iPSCs after 5 and 25 passages. **f)** iPSC marker expression after 30 passages. **g)** Differentiation of iPSCs into ectoderm, endoderm, and mesoderm lineages. **h)** Embryoid body generated from iPSCs. **i)** Differentiation of late-passage (30 passages) into ectoderm, endoderm, and mesoderm lineages. **j)** Cardiac marker expression in iPSC-derived cardiac organoids. **k)** RT-PCR confirmation of cardiac marker expression in iPSC-derived cardiac organoids. Data in **b, k** are representative of *n* = 3 biologically independent experiments. Error bars in **e** represent standard deviation for *n* = 3 replicates. Scale bars in **a, c, f, g, i, j** represent 100um.

**Supplementary Figure 3. Profiling activity of existing ASO therapeutics in patient-derived organoid model of disease. a)** Dystrophin expression in DMD patient-derived cardiac organoids generated from patient 1 using alternative differentiation protocol and treated with ASOs matching the sequence of existing ASO therapeutics. **b)** Quantification of DMD expression in DMD patient-derived cardiac organoids (patient 1) generated using alternative differentiation protocol and treated with ASOs matching the sequence of existing ASO therapeutics. **c)** Contraction of individual DMD patient-derived cardiac organoids (patient 1) generated using alternative differentiation protocol and treated with ASOs matching the sequence of existing ASO therapeutics. Error bars in **b** represent standard deviation for *n* = 3 replicates. Scale bars in **a** represent 100um.

**Supplementary Figure 4. Profiling activity of existing ASO therapeutics in patient-derived skeletal muscle. a)** Dystrophin expression in DMD patient-derived skeletal muscle generated from patient 1 treated with ASOs matching the sequence of existing ASO therapeutics. **b)** Quantification of DMD expression in DMD patient-derived skeletal muscle (patient 1) treated with ASOs matching the sequence of existing ASO therapeutics. Error bars in **b** represent standard deviation for *n* = 3 replicates. Scale bars in **a** represent 100um.

**Supplementary Figure 5. Z-stack analysis of cardiac organoids treated with patient-specific ASOs.** **a)** Z-stack analysis of dystrophin expression in DMD patient-derived cardiac organoids treated with patient-specific ASOs. Scale bars in **a** represent 100um.

**Supplementary Figure 6. Profiling activity of patient-specific ASOs in patient-derived organoid model of disease. a)** Dystrophin expression in DMD patient-derived cardiac organoids generated from patient 2a using alternative differentiation protocol and treated with patient-specific ASOs. **b)** Quantification of DMD expression in DMD patient-derived cardiac organoids (patient 2a) generated using alternative differentiation protocol and treated with patient-specific ASOs. **c)** Contraction of individual DMD patient-derived cardiac organoids (patient 2a) generated using alternative differentiation protocol and treated with patient-specific ASOs. Error bars in **b** represent standard deviation for *n* = 3 replicates. Scale bars in **a** represent 100um.

**Supplementary Figure 7. Profiling activity of patient-specific ASOs in patient-derived skeletal muscle. a)** Skeletal muscle marker expression in patient-derived skeletal muscle (patient 2a). **b)** Dystrophin expression in DMD patient-derived skeletal muscle generated from patient 2a treated with patient-specific ASOs. **c)** Quantification of DMD expression in DMD patient-derived skeletal muscle (patient 2a) treated with patient-specific ASOs. Error bars in **c** represent standard deviation for *n* = 3 replicates. Scale bars in **a**,**b** represent 100um.

**Supplementary Figure 8. Profiling activity of patient-specific ASOs in patient-derived organoid model of disease. a)** Dystrophin expression in DMD patient-derived cardiac organoids generated from patient 2b using alternative differentiation protocol and treated with patient-specific ASOs. **b)** Quantification of DMD expression in DMD patient-derived cardiac organoids (patient 2b) generated using alternative differentiation protocol and treated with patient-specific ASOs. **c)** Contraction of individual DMD patient-derived cardiac organoids (patient 2b) generated using alternative differentiation protocol and treated with patient-specific ASOs. Error bars in **b** represent standard deviation for *n* = 3 replicates. Scale bars in **a** represent 100um.

**Supplementary Figure 9. Profiling activity of patient-specific ASOs in patient-derived skeletal muscle. a)** Skeletal muscle marker expression in patient-derived skeletal muscle (patient 2b). **b)** Dystrophin expression in DMD patient-derived skeletal muscle generated from patient 2b treated with patient-specific ASOs. **c)** Quantification of DMD expression in DMD patient-derived skeletal muscle (patient 2b) treated with patient-specific ASOs. Error bars in **c** represent standard deviation for *n* = 3 replicates. Scale bars in **a**,**b** represent 100um.

**Supplementary Figure 10. Chamber formation in patient-derived cardiac organoids. a)** Representative images of patient-derived organoids with chamber structures. **b)** Representative images of patient-derived organoids generated using alternative differentiation protocol with chamber structures.

**Supplementary Figure 11.** **Profiling activity of patient-specific ASOs in patient-derived brain organoids. a)** Neuronal marker expression in patient-derived cerebral organoids (patient 2a). **b)** RT-PCR confirmation of neuronal marker expression in patient-derived cerebral organoids (patient 2a). **c)** Dystrophin expression in DMD patient-derived cerebral organoids (patient 2a) treated with patient-specific ASOs. **d)** Quantification of DMD expression in DMD patient-derived cerebral organoids (patient 2a) treated with patient-specific ASOs. **e)** Efficiency of ASO transfection in patient-derived cerebral organoids (patient 2a). Data in **b** are representative of *n* = 3 biologically independent experiments. Error bars in **d, e** represent standard deviation for *n* = 3 replicates. Scale bars in **a**, **c** represent 100um.

**Supplementary Figure 12.** **Individual flow cytometry analyses.** Individual flow cytometry analyses.

**Supplementary Figure 13. Uncropped scans of western blots and agarose gels.** Uncropped scans of western blots and agarose gels**.**
